# Risk stratification of patients admitted to hospital with covid-19 using the ISARIC WHO Clinical Characterisation Protocol: development and validation of the 4C Mortality Score

**DOI:** 10.1136/bmj.m3339

**Published:** 2020-09-09

**Authors:** Stephen R Knight, Antonia Ho, Riinu Pius, Iain Buchan, Gail Carson, Thomas M Drake, Jake Dunning, Cameron J Fairfield, Carrol Gamble, Christopher A Green, Rishi Gupta, Sophie Halpin, Hayley E Hardwick, Karl A Holden, Peter W Horby, Clare Jackson, Kenneth A Mclean, Laura Merson, Jonathan S Nguyen-Van-Tam, Lisa Norman, Mahdad Noursadeghi, Piero L Olliaro, Mark G Pritchard, Clark D Russell, Catherine A Shaw, Aziz Sheikh, Tom Solomon, Cathie Sudlow, Olivia V Swann, Lance CW Turtle, Peter JM Openshaw, J Kenneth Baillie, Malcolm G Semple, Annemarie B Docherty, Ewen M Harrison, ISARIC4C investigators, J Kenneth Baillie, Malcolm G Semple, Peter JM Openshaw, Gail Carson, Beatrice Alex, Benjamin Bach, Wendy S Barclay, Debby Bogaert, Meera Chand, Graham S Cooke, Annemarie B Docherty, Jake Dunning, Ana da Silva Filipe, Tom Fletcher, Christopher A Green, Ewen M Harrison, Julian A Hiscox, Antonia Ying Wai Ho, Peter W Horby, Samreen Ijaz, Saye Khoo, Paul Klenerman, Andrew Law, Wei Shen Lim, Alexander J Mentzer, Laura Merson, Alison M Meynert, Mahdad Noursadeghi, Shona C Moore, Massimo Palmarini, William A Paxton, Georgios Pollakis, Nicholas Price, Andrew Rambaut, David L Robertson, Clark D Russell, Vanessa Sancho-Shimizu, Janet T Scott, Louise Sigfrid, Tom Solomon, Shiranee Sriskandan, David Stuart, Charlotte Summers, Richard S Tedder, Emma C Thomson, Ryan S Thwaites, Lance CW Turtle, Maria Zambon, Hayley Hardwick, Chloe Donohue, Jane Ewins, Wilna Oosthuyzen, Fiona Griffiths, Lisa Norman, Riinu Pius, Tom M Drake, Cameron J Fairfield, Stephen Knight, Kenneth A Mclean, Derek Murphy, Catherine A Shaw, Jo Dalton, Michelle Girvan, Egle Saviciute, Stephanie Roberts, Janet Harrison, Laura Marsh, Marie Connor, Sophie Halpin, Clare Jackson, Carrol Gamble, Gary Leeming, Andrew Law, Ross Hendry, James Scott-Brown, William Greenhalf, Victoria Shaw, Sarah McDonald, Katie A Ahmed, Jane A Armstrong, Milton Ashworth, Innocent G Asiimwe, Siddharth Bakshi, Samantha L Barlow, Laura Booth, Benjamin Brennan, Katie Bullock, Benjamin WA Catterall, Jordan J Clark, Emily A Clarke, Sarah Cole, Louise Cooper, Helen Cox, Christopher Davis, Oslem Dincarslan, Chris Dunn, Philip Dyer, Angela Elliott, Anthony Evans, Lewis WS Fisher, Terry Foster, Isabel Garcia-Dorival, Willliam Greenhalf, Philip Gunning, Catherine Hartley, Antonia Ho, Rebecca L Jensen, Christopher B Jones, Trevor R Jones, Shadia Khandaker, Katharine King, Robyn T Kiy, Chrysa Koukorava, Annette Lake, Suzannah Lant, Diane Latawiec, L Lavelle-Langham, Daniella Lefteri, Lauren Lett, Lucia A Livoti, Maria Mancini, Sarah McDonald, Laurence McEvoy, John McLauchlan, Soeren Metelmann, Nahida S Miah, Joanna Middleton, Joyce Mitchell, Shona C Moore, Ellen G Murphy, Rebekah Penrice-Randal, Jack Pilgrim, Tessa Prince, Will Reynolds, P Matthew Ridley, Debby Sales, Victoria E Shaw, Rebecca K Shears, Benjamin Small, Krishanthi S Subramaniam, Agnieska Szemiel, Aislynn Taggart, Jolanta Tanianis-Hughes, Jordan Thomas, Erwan Trochu, Libby van Tonder, Eve Wilcock, J Eunice Zhang, Kayode Adeniji, Daniel Agranoff, Ken Agwuh, Dhiraj Ail, Ana Alegria, Brian Angus, Abdul Ashish, Dougal Atkinson, Shahedal Bari, Gavin Barlow, Stella Barnass, Nicholas Barrett, Christopher Bassford, David Baxter, Michael Beadsworth, Jolanta Bernatoniene, John Berridge, Nicola Best, Pieter Bothma, David Brealey, Robin Brittain-Long, Naomi Bulteel, Tom Burden, Andrew Burtenshaw, Vikki Caruth, David Chadwick, Duncan Chambler, Nigel Chee, Jenny Child, Srikanth Chukkambotla, Tom Clark, Paul Collini, Catherine Cosgrove, Jason Cupitt, Maria-Teresa Cutino-Moguel, Paul Dark, Chris Dawson, Samir Dervisevic, Phil Donnison, Sam Douthwaite, Ingrid DuRand, Ahilanadan Dushianthan, Tristan Dyer, Cariad Evans, Chi Eziefula, Chrisopher Fegan, Adam Finn, Duncan Fullerton, Sanjeev Garg, Sanjeev Garg, Atul Garg, Jo Godden, Arthur Goldsmith, Clive Graham, Elaine Hardy, Stuart Hartshorn, Daniel Harvey, Peter Havalda, Daniel B Hawcutt, Maria Hobrok, Luke Hodgson, Anita Holme, Anil Hormis, Michael Jacobs, Susan Jain, Paul Jennings, Agilan Kaliappan, Vidya Kasipandian, Stephen Kegg, Michael Kelsey, Jason Kendall, Caroline Kerrison, Ian Kerslake, Oliver Koch, Gouri Koduri, George Koshy, Shondipon Laha, Susan Larkin, Tamas Leiner, Patrick Lillie, James Limb, Vanessa Linnett, Jeff Little, Michael MacMahon, Emily MacNaughton, Ravish Mankregod, Huw Masson, Elijah Matovu, Katherine McCullough, Ruth McEwen, Manjula Meda, Gary Mills, Jane Minton, Mariyam Mirfenderesky, Kavya Mohandas, Quen Mok, James Moon, Elinoor Moore, Patrick Morgan, Craig Morris, Katherine Mortimore, Samuel Moses, Mbiye Mpenge, Rohinton Mulla, Michael Murphy, Megan Nagel, Thapas Nagarajan, Mark Nelson, Igor Otahal, Mark Pais, Selva Panchatsharam, Hassan Paraiso, Brij Patel, Justin Pepperell, Mark Peters, Mandeep Phull, Stefania Pintus, Jagtur Singh Pooni, Frank Post, David Price, Rachel Prout, Nikolas Rae, Henrik Reschreiter, Tim Reynolds, Neil Richardson, Mark Roberts, Devender Roberts, Alistair Rose, Guy Rousseau, Brendan Ryan, Taranprit Saluja, Aarti Shah, Prad Shanmuga, Anil Sharma, Anna Shawcross, Jeremy Sizer, Richard Smith, Catherine Snelson, Nick Spittle, Nikki Staines, Tom Stambach, Richard Stewart, Pradeep Subudhi, Tamas Szakmany, Kate Tatham, Jo Thomas, Chris Thompson, Robert Thompson, Ascanio Tridente, Darell Tupper-Carey, Mary Twagira, Andrew Ustianowski, Nick Vallotton, Lisa Vincent-Smith, Shico Visuvanathan, Alan Vuylsteke, Sam Waddy, Rachel Wake, Andrew Walden, Ingeborg Welters, Tony Whitehouse, Paul Whittaker, Ashley Whittington, Meme Wijesinghe, Martin Williams, Lawrence Wilson, Sarah Wilson, Stephen Winchester, Martin Wiselka, Adam Wolverson, Daniel G Wooton, Andrew Workman, Bryan Yates, Peter Young

**Affiliations:** 1 Centre for Medical Informatics, The Usher Institute, University of Edinburgh, Edinburgh, UK; 2 Medical Research Council, University of Glasgow Centre for Virus Research, Glasgow, UK; 3 Department of Infectious Diseases, Queen Elizabeth University Hospital, Glasgow, UK; 4 Institute of Population Health Sciences, University of Liverpool, Liverpool, UK; 5 ISARIC Global Support Centre, Centre for Tropical Medicine and Global Health, Nuffield Department of Medicine, University of Oxford, Oxford, UK; 6 National Infection Service, Public Health England, London, UK; 7 National Heart and Lung Institute, Imperial College London, London, UK; 8 Liverpool Clinical Trials Centre, University of Liverpool, Liverpool, UK; 9 Institute of Microbiology & Infection, University of Birmingham, Birmingham, UK; 10 Institute of Global Health, University College London, London, UK; 11 NIHR Health Protection Research Unit, Institute of Infection, Veterinary and Ecological Sciences, Faculty of Health and Life Sciences, University of Liverpool, Liverpool, UK; 12 Division of Epidemiology and Public Health, University of Nottingham School of Medicine, Nottingham, UK; 13 Division of Infection and Immunity, University College London, London, UK; 14 Centre for Tropical Medicine and Global Health, Nuffield Department of Medicine, University of Oxford, Oxford, UK; 15 Queen’s Medical Research Institute, University of Edinburgh, Edinburgh, UK; 16 Walton Centre NHS Foundation Trust, Liverpool, UK; 17 Health Data Research UK, London, UK; 18 Department of Child Life and Health, University of Edinburgh, Edinburgh, UK; 19 Tropical & Infectious Disease Unit, Royal Liverpool University Hospital, Liverpool, UK; 20 Roslin Institute, University of Edinburgh, Edinburgh, UK; 21 Intensive Care Unit, Royal Infirmary Edinburgh, Edinburgh, UK; 22 Respiratory Medicine, Alder Hey Children’s Hospital, Institute in The Park, University of Liverpool, Alder Hey Children’s Hospital, Liverpool L12 2AP, UK; 23 Department of Clinical Surgery, University of Edinburgh, Edinburgh, UK

## Abstract

**Objective:**

To develop and validate a pragmatic risk score to predict mortality in patients admitted to hospital with coronavirus disease 2019 (covid-19).

**Design:**

Prospective observational cohort study.

**Setting:**

International Severe Acute Respiratory and emerging Infections Consortium (ISARIC) World Health Organization (WHO) Clinical Characterisation Protocol UK (CCP-UK) study (performed by the ISARIC Coronavirus Clinical Characterisation Consortium—ISARIC-4C) in 260 hospitals across England, Scotland, and Wales. Model training was performed on a cohort of patients recruited between 6 February and 20 May 2020, with validation conducted on a second cohort of patients recruited after model development between 21 May and 29 June 2020*.*

**Participants:**

Adults (age ≥18 years) admitted to hospital with covid-19 at least four weeks before final data extraction.

**Main outcome measure:**

In-hospital mortality.

**Results:**

35 463 patients were included in the derivation dataset (mortality rate 32.2%) and 22 361 in the validation dataset (mortality rate 30.1%). The final 4C Mortality Score included eight variables readily available at initial hospital assessment: age, sex, number of comorbidities, respiratory rate, peripheral oxygen saturation, level of consciousness, urea level, and C reactive protein (score range 0-21 points). The 4C Score showed high discrimination for mortality (derivation cohort: area under the receiver operating characteristic curve 0.79, 95% confidence interval 0.78 to 0.79; validation cohort: 0.77, 0.76 to 0.77) with excellent calibration (validation: calibration-in-the-large=0, slope=1.0). Patients with a score of at least 15 (n=4158, 19%) had a 62% mortality (positive predictive value 62%) compared with 1% mortality for those with a score of 3 or less (n=1650, 7%; negative predictive value 99%). Discriminatory performance was higher than 15 pre-existing risk stratification scores (area under the receiver operating characteristic curve range 0.61-0.76), with scores developed in other covid-19 cohorts often performing poorly (range 0.63-0.73).

**Conclusions:**

An easy-to-use risk stratification score has been developed and validated based on commonly available parameters at hospital presentation. The 4C Mortality Score outperformed existing scores, showed utility to directly inform clinical decision making, and can be used to stratify patients admitted to hospital with covid-19 into different management groups. The score should be further validated to determine its applicability in other populations.

**Study registration:**

ISRCTN66726260

## Introduction

Disease resulting from infection with severe acute respiratory syndrome coronavirus 2 (SARS-CoV-2) has a high mortality rate with deaths predominantly caused by respiratory failure.^1^ As of 1 September 2020, over 25 million people had confirmed coronavirus disease 2019 (covid-19) worldwide and at least 850 000 people had died from the disease.^2^ ^3^ As hospitals around the world are faced with an influx of patients with covid-19, there is an urgent need for a pragmatic risk stratification tool that will allow the early identification of patients infected with SARS-CoV-2 who are at the highest risk of death to guide management and optimise resource allocation.

Prognostic scores attempt to transform complex clinical pictures into tangible numerical values. Prognostication is more difficult when dealing with a severe pandemic illness such as covid-19 because strain on healthcare resources and rapidly evolving treatments alter the risk of death over time. Early information has suggested that the clinical course of a patient with covid-19 is different from that of pneumonia, seasonal influenza, or sepsis.^4^ Most patients with severe covid-19 have developed a clinical picture characterised by pneumonitis, profound hypoxia, and systemic inflammation affecting multiple organs.^1^

A recent review identified many prognostic scores used for covid-19,^5^ which varied in their setting, predicted outcome measure, and the clinical parameters included. The large number of risk stratification tools reflects difficulties in their application, with most scores showing moderate performance at best and no benefit to clinical decision making.^6^ ^7^ Many novel covid-19 prognostic scores have been found to have a high risk of bias, which could reflect development in small cohorts, and many have been published without clear details of model derivation and testing.^5^ Therefore, a risk stratification tool within a large national cohort of patients admitted to hospital with covid-19 is needed with clear development and validation details.

Our aim was to develop and validate a pragmatic, clinically relevant risk stratification score that uses routinely available clinical information at hospital presentation to predict in-hospital mortality in patients admitted to hospital with covid-19. We then aimed to compare this score with existing prognostic models.

## Methods

### Study design and setting

The International Severe Acute Respiratory and emerging Infections Consortium (ISARIC) World Health Organization (WHO) Clinical Characterisation Protocol UK (CCP-UK) study is an ongoing prospective cohort study. The study is being performed by the ISARIC Coronavirus Clinical Characterisation Consortium (ISARIC-4C) in 260 hospitals across England, Scotland, and Wales (National Institute for Health Research Clinical Research Network Central Portfolio Management System ID 14152). The protocol and further study details are available online.^8^ Model development and reporting followed the TRIPOD (transparent reporting of a multivariable prediction model for individual prediction or diagnosis) guidelines.^9^ The study is being conducted according to a predefined protocol (appendix 1).

### Participants

The study recruited consecutive patients aged 18 years and older with a completed index admission to one of 260 hospitals in England, Scotland, or Wales.^8^ Reverse transcriptase polymerase chain reaction was the only mode of testing available during the period of study. The decision to test was at the discretion of the clinician attending the patient, and not defined by protocol. The enrolment criterion “high likelihood of infection” reflected that a preparedness protocol cannot assume a diagnostic test will be available for an emergent pathogen. In this activation, site training emphasised the importance of only recruiting proven cases.

### Data collection

Demographic, clinical, and outcome data were collected by using a prespecified case report form. Comorbidities were defined according to a modified Charlson comorbidity index.^10^ Comorbidities collected were chronic cardiac disease, chronic respiratory disease (excluding asthma), chronic renal disease (estimated glomerular filtration rate ≤30), mild to severe liver disease, dementia, chronic neurological conditions, connective tissue disease, diabetes mellitus (diet, tablet, or insulin controlled), HIV or AIDS, and malignancy. These conditions were selected a priori by a global consortium to provide rapid, coordinated clinical investigation of patients presenting with any severe or potentially severe acute infection of public interest and enabled standardisation.

Clinician defined obesity was also included as a comorbidity owing to its probable association with adverse outcomes in patients with covid-19.^11^ ^12^ The clinical information used to calculate prognostic scores was taken from the day of admission to hospital.^13^ A practical approach was taken to sample size requirements.^14^ We used all available data to maximise the power and generalisability of our results. Model reliability was assessed by using a temporally distinct validation cohort with geographical subsetting, together with sensitivity analyses.

### Outcomes

The primary outcome was in-hospital mortality. This outcome was selected because of the importance of the early identification of patients likely to develop severe illness from SARS-CoV-2 infection (a rule in test). We chose to restrict analysis of outcomes to patients who were admitted more than four weeks before final data extraction (29 June 2020) to enable most patients to complete their hospital admission.

### Independent predictor variables

A reduced set of potential predictor variables was selected a priori, including patient demographic information, common clinical investigations, and parameters consistently identified as clinically important in covid-19 cohorts following the methods described by Wynants and colleagues (appendix 2).^5^ Candidate predictor variables were selected based on three common criteria^15^: patient and clinical variables known to influence outcome in pneumonia and flu-like illness; clinical biomarkers previously identified within the literature as potential predictors in patients with covid-19; values available for at least two thirds of patients within the derivation cohort.

Because our overall aim was to develop an easy-to-use risk stratification score, we made the decision to include an overall comorbidity count for each patient within model development giving each comorbidity equal weight, rather than individual comorbidities. Recent evidence suggests an additive effect of comorbidity in patients with covid-19, with increasing number of comorbidities associated with poorer outcomes.^16^

### Model development

Missing values for potential candidate variables were handled by using multiple imputation with chained equations, under the missing at random assumption (appendix 6). Ten sets, each with 10 iterations, were imputed using available explanatory variables for both cohorts (derivation and validation). The outcome variable was included as a predictor in the derivation dataset but not the validation dataset. All model derivation and validation was performed in imputed datasets, with Rubin’s rules^17^ used to combine results.

Models were trained by using all available data up to 20 May 2020. The primary intention was to create a pragmatic model for bedside use not requiring complex equations, online calculators, or mobile applications. An a priori decision was therefore made to categorise continuous variables in the final prognostic score.

**Fig 1:**
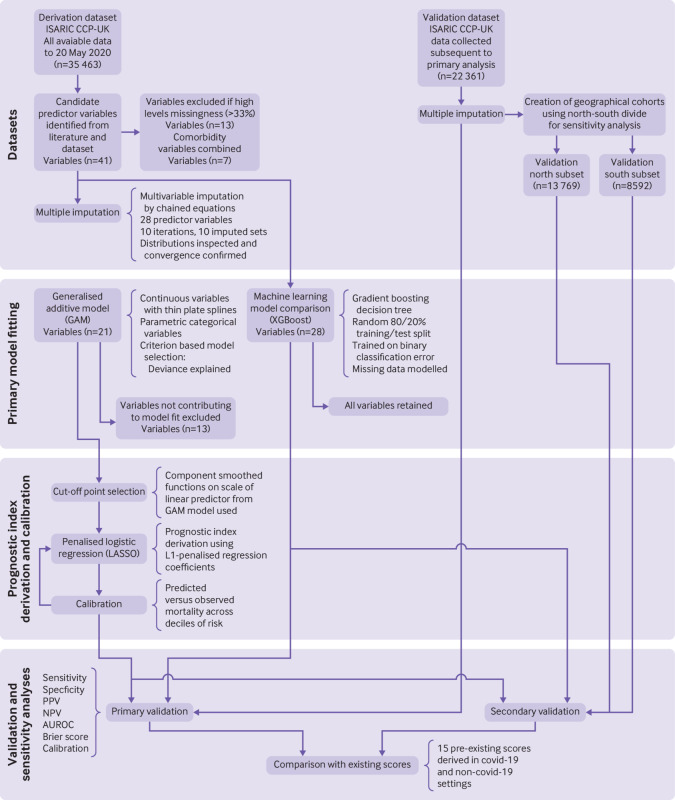
Model derivation and validation workflow. AUROC=area under the receiver operating characteristic curve; covid-19=coronavirus disease 2019; ISARIC CCP-UK=International Severe Acute Respiratory and emerging Infections Consortium Clinical Characterisation Protocol UK; NPV=negative predictive value; PPV=positive predictive value

We used a three stage model building process (fig 1). Firstly, generalised additive models were built incorporating continuous smoothed predictors (penalised thin plate splines) in combination with categorical predictors as linear components. A criterion based approach to variable selection was taken based on the deviance explained, the unbiased risk estimator, and the area under the receiver operating characteristic curve. Secondly, we visually inspected plots of component smoothed continuous predictors for linearity, and selected optimal cut-off values by using the methods of Barrio and colleagues.^18^

Lastly, final models using categorised variables were specified with least absolute shrinkage and selection operator logistic regression. L1 penalised coefficients were derived using 10-fold cross validation to select the value of lambda (minimised cross validated sum of squared residuals). We converted shrunk coefficients to a prognostic index with appropriate scaling to create the pragmatic 4C Mortality Score (where 4C stands for Coronavirus Clinical Characterisation Consortium).

We used machine learning approaches in parallel for comparison of predictive performance. Given issues with interpretability, this was intended to provide a best-in-class comparison of predictive performance when accounting for any complex underlying interactions. Gradient boosting decision trees were used (XGBoost). All candidate predictor variables identified were included within the model, except for those with high missing values (>33%). We retained individual major comorbidity variables within the model to determine whether inclusion improved predictive performance. An 80%/20% random split of the derivation dataset was used to define train and test sets. The validation datasets were held back and not used in the training process. We used a mortality label and design matrix of centred or standardised continuous and categorical variables including all candidate variables to train gradient boosted trees minimising the binary classification error rate (defined as number of wrong cases divided by number of all cases). Hyperparameters were tuned, including the learning rate and maximum tree depth, to maximise the area under the receiver operating characteristic curve in the test set. This approach affords flexibility in the handling of missing data; therefore, two models were trained and optimised, one using imputed data and the other modelling missingness in complete case data.

We assessed discrimination for all models by using the area under the receiver operating characteristic curve in the derivation cohort, with 95% confidence intervals calculated by bootstrapped resampling (2000 samples). A value of 0.5 indicates no predictive ability, 0.8 is considered good, and 1.0 is perfect.^19^ We assessed overall goodness of fit with the Brier score,^20^ a measure to quantify how close predictions are to the truth. The score ranges between 0 and 1, where smaller values indicate superior model performance. We plotted model calibration curves to examine agreement between predicted and observed risk across deciles of mortality risk to determine the presence of over or under prediction. Risk cut-off values were defined by the total point score for an individual, which represented low (<2% mortality rate), intermediate (2-14.9%), or high risk (≥15%) groups, similar to commonly used pneumonia risk stratification scores.^21^ ^22^

We performed sensitivity analyses by using complete case data. Model discrimination was also checked in ethnic groups and by sex using imputed datasets.

### Model validation

Patients entered into the ISARIC WHO CCP-UK study after 20 May 2020 were included in a separate validation cohort (fig 1). We determined discrimination, calibration, and performance across a range of clinically relevant metrics. To avoid bias in the assessment of outcomes, patients who were admitted within four weeks of data extraction on 29 June 2020 were excluded. We included patients without an outcome after four weeks and considered to have had no event.

A sensitivity analysis was also performed, with stratification of the validation cohort by geographical location. We selected this geographical categorisation based on well described economic and health inequalities between the north and south of the United Kingdom.^23^ ^24^ Recent analysis has shown the impact of deprivation on risk of dying with covid-19.^25^ As a result, population differences between regions could change the discriminatory performance of risk stratification scores. Two geographical cohorts were created, based on north-south geographical locations across the UK as defined by Hacking and colleagues.^23^ We performed a further sensitivity analysis to determine model performance in ethnic minority groups given the reported differences in covid-19 outcomes.^26^

All tests were two tailed and P values less than 0.05 were considered statistically significant. We used R (version 3.6.3) with the finalfit, mice, glmnet, pROC, recipes, xgboost, rmda, and tidyverse packages for all statistical analysis.

### Comparison with existing risk stratification scores

All derived models in the derivation dataset were compared within the validation cohort with existing scores. We assessed model performance by using the area under the receiver operating characteristic curve statistic, sensitivity, specificity, positive predictive value, and negative predictive value. Existing risk stratification scores were identified through a systematic literature search of Embase, WHO Medicus, and Google Scholar databases. We used the search terms “pneumonia,” “sepsis,” “influenza,” “COVID-19,” “SARS-CoV-2,” “coronavirus” combined with “score” and “prognosis.” We applied no language or date restrictions. The last search was performed on 1 July 2020. Risk stratification tools were included whose variables were available within the database and had accessible methods for calculation.

We calculated performance characteristics according to original publications, and selected score cut-off values for adverse outcomes based on the most commonly used criteria identified within the literature. Cut-off values were the score value for which the patient was considered at low or high risk of adverse outcome, as defined by the study authors. Patients with one or more missing input variables were omitted for that particular score.

We also performed a decision curve analysis.^27^ Briefly, assessment of the adequacy of clinical prediction models can be extended by determining clinical utility. By using decision curve analysis, we can make a clinical judgment about the relative value of benefits (treating a true positive) and harms (treating a false positive) associated with a clinical prediction tool. The standardised net benefit was plotted against the threshold probability for considering a patient high risk for age alone and for the best discriminating models applicable to more than 50% of patients in the validation cohort.

### Patient and public involvement

This was an urgent public health research study in response to a Public Health Emergency of International Concern. Patients or the public were not involved in the design, conduct, or reporting of this rapid response research.

## Results

We collected data from 35 463 patients between 6 February 2020 and 20 May 2020 in the derivation cohort; 1275 (3.6%) patients had no outcome recorded and were considered alive. The overall mortality rate was 32.2% (11 426 patients). The median age of patients in the cohort was 73 years (interquartile range 59-83); 41.7% (14 741) were female and 76.0% (26 966) had at least one comorbidity. Table 1 shows demographic and clinical characteristics for the derivation and validation datasets.

**Table 1:** Demographic and clinical characteristics for derivation and validation cohorts of patients admitted to hospital with covid-19

Characteristics	Derivation cohort			Validation cohort	
	No of patients (%) or median (IQR)	Total No (%)		No of patients (%) or median (IQR)	Total No (%)
Mortality in hospital	11 426 (32.2)	35 463 (100.0)		6729 (30.1)	22 361 (100.0)
Age (years)					
<50	4876 (13.8)	35 277 (99.5)		2808 (12.6)	22 361 (100.0)
50-69	10 183 (28.9)	—		5762 (25.8)	—
70-79	8017 (22.7)	—		4951 (22.1)	—
≥80	12 201 (34.6)	—		8840 (39.5)	—
Sex at birth					
Female	14 741 (41.7)	35 356 (99.7)		10 178 (45.6)	22 319 (99.8)
Ethnicity					
White	26 300 (82.2)	31 987 (90.2)		16 831 (84.9)	19 818 (88.6)
South Asian	1647 (5.1)	—		811 (4.1)	—
East Asian	271 (0.8)	—		140 (0.7)	—
Black	1256 (3.9)	—		769 (3.9)	—
Other ethnic minority	2513 (7.9)	—		1267 (6.4)	—
Chronic cardiac disease	10 513 (31.8)	33 090 (93.3)		7019 (34.0)	20 616 (92.2)
Chronic kidney disease	5653 (17.2)	32 834 (92.6)		3769 (18.4)	20 444 (91.4)
Malignant neoplasm	3312 (10.2)	32 556 (91.8)		2187 (10.8)	20 297 (90.8)
Moderate or severe liver disease	604 (1.9)	32 538 (91.8)		434 (2.1)	20 218 (90.4)
Obesity (clinician defined)	3414 (11.4)	29 829 (84.1)		2234 (12.2)	18 304 (81.9)
Chronic pulmonary disease (not asthma)	5830 (17.7)	32 990 (93.0)		3737 (18.2)	20 502 (91.7)
Diabetes (type 1 and 2)	8487 (26.0)	32 622 (92.0)		4275 (21.9)	19 511 (87.3)
No of comorbidities					
0	8497 (24.0)	35 463 (100.0)		5098 (22.8)	22 361 (100.0)
1	9941 (28.0)	—		6114 (27.3)	—
≥2	17 025 (48.0)	—		11 149 (49.9)	—
Respiratory rate (breaths/min)	22.0 (9.0)	33 330 (94.0)		20.0 (8.0)	20 970 (93.8)
Oxygen saturation (%)	94.0 (6.0)	33 696 (95.0)		94.0 (5.0)	21 197 (94.8)
Systolic blood pressure (mm Hg)	124.0 (33.0)	33 637 (94.9)		129.0 (33.0)	21 073 (94.2)
Diastolic blood pressure (mm Hg)	70.0 (19.0)	33 568 (94.7)		73.0 (20.0)	21 026 (94.0)
Temperature (ºC)	37.3 (1.5)	33 467 (94.4)		37.1 (1.5)	21 139 (94.5)
Heart rate (bpm)	90.0 (27.0)	33 405 (94.2)		90.0 (28.0)	20 991 (93.9)
Glasgow coma scale score	15.0 (0.0)	30 819 (86.9)		15.0 (0.0)	20 015 (89.5)
Haemoglobin (g/L)	129.0 (30.0)	29 924 (84.4)		127.0 (31.0)	18 480 (82.6)
White blood cell count (10^9^/L)	7.4 (5.1)	29 740 (83.9)		7.6 (5.3)	18 362 (82.1)
Neutrophil count (10^9^/L)	5.6 (4.6)	29 594 (83.5)		5.8 (4.9)	18 354 (82.1)
Lymphocyte count (10^9^/L)	0.9 (0.7)	29 553 (83.3)		0.9 (0.7)	18 348 (82.1)
Platelet count (10^9^/L)	216.0 (120.0)	29 582 (83.4)		223.0 (126.0)	18 281 (81.8)
Sodium (mmol/L)	137.0 (6.0)	29 522 (83.2)		137.0 (6.0)	18 409 (82.3)
Potassium (mmol/L)	4.1 (0.8)	27 224 (76.8)		4.1 (0.8)	16 926 (75.7)
Total bilirubin (mg/dL)	10.0 (7.0)	24 446 (68.9)		10.0 (7.0)	15 404 (68.9)
Urea (mmol/L)	7.0 (6.3)	26 122 (73.7)		7.3 (6.8)	16 863 (75.4)
Creatinine (μmol/L**)**	86.0 (53.0)	29 439 (83.0)		86.0 (56.0)	18 225 (81.5)
C reactive protein (mg/L)	84.9 (122.0)	27 856 (78.5)		78.0 (120.0)	17 119 (76.6)

Covid-19=coronavirus disease 2019; IQR=interquartile range.

Comorbidities were defined using the Charlson comorbidity index, with the addition of clinician defined obesity.

### Model development

We identified 41 candidate predictor variables measured at hospital admission for model creation (fig 1, appendix 2). After the creation of a composite variable containing all seven individual comorbidities and the exclusion of 13 variables owing to high levels of missing values, 21 variables remained.

We identified eight important predictors of mortality by using generalised additive modelling with multiply imputed datasets: age, sex, number of comorbidities, respiratory rate, peripheral oxygen saturation, Glasgow coma scale, urea level, and C reactive protein (for variable selection process, see appendix 3). Given the need for a pragmatic score for use at the bedside, continuous variables were converted to factors with cut-off values chosen by using component smoothed functions (on linear predictor scale) from generalised additive modelling (appendix 4).

On entering variables into a penalised logistic regression model (least absolute shrinkage and selection operator), all variables were retained within the final model (appendix 5). We converted penalised regression coefficients into a prognostic index by using appropriate scaling (4C Mortality Score range 0-21 points; table 2).

**Table 2:** Final 4C Mortality Score for in-hospital mortality in patients with covid-19. Prognostic index derived from penalised logistic regression (LASSO) model

Variable	4C Mortality Score
Age (years)	
<50	—
50-59	+2
60-69	+4
70-79	+6
≥80	+7
Sex at birth	
Female	—
Male	+1
No of comorbidities*	
0	—
1	+1
≥2	+2
Respiratory rate (breaths/min)	
<20	—
20-29	+1
≥30	+2
Peripheral oxygen saturation on room air (%)	
≥92	—
<92	+2
Glasgow coma scale score	
15	—
<15	+2
Urea (mmol/L)	
<7	—
7-14	+1
>14	+3
C reactive protein (mg/L)	
<50	—
50-99	+1
≥100	+2

Covid-19=coronavirus disease 2019.

* Comorbidities were defined by using Charlson comorbidity index, with the addition of clinician defined obesity.

The 4C Mortality Score showed good discrimination for death in hospital within the derivation cohort (table 3), with performance approaching that of the XGBoost model. The 4C Mortality Score showed good calibration (calibration intercept=0, slope=1, Brier score 0.170) across the range of risk and no adjustment to the model was required (appendix 11).

**Table 3:** Model discrimination in derivation and validation cohorts

Model	Derivation cohort			Validation cohort	
	AUROC (95% CI)	Brier score		AUROC (95% CI)	Brier score
4C Mortality Score	0.786 (0.781 to 0.790)	0.170		0.767 (0.760 to 0.773)	0.171
Machine learning comparison*	0.796 (0.786 to 0.807)	0.191		0.779 (0.772 to 0.785)	0.197

AUROC=area under receiver operator curve; CI=confidence interval.

* Gradient boosting decision tree (XGBoost).

### Model validation

The validation cohort included data from 22 361 patients collected between 21 May 2020 and 29 June 2020 who had at least four weeks of follow-up; 743 (3.3%) patients had no outcome recorded and were considered alive. The overall mortality rate was 30.1% (6729 patients). The median age of patients in the cohort was 76 (interquartile range 60-85) years; 10 178 (45.6%) were female and 17 263 (77%) had at least one comorbidity (table 1).

**Fig 2:**
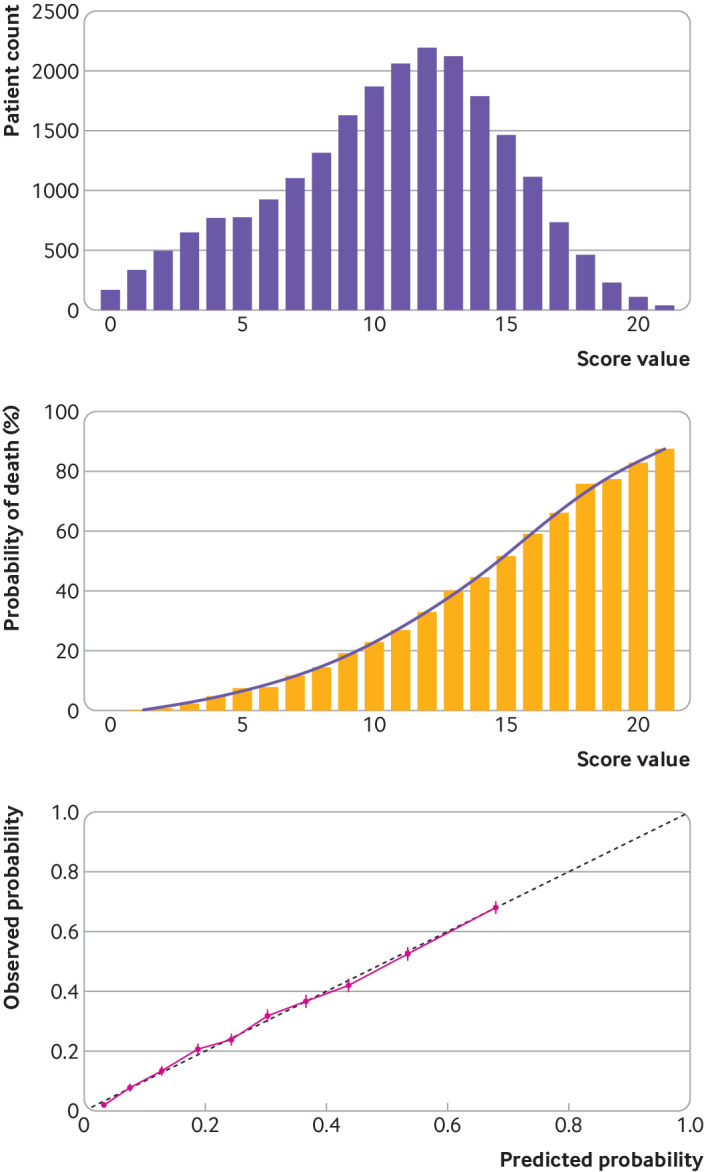
Upper panel: distribution of patients across range of 4C Mortality Score in validation cohort; middle panel: observed in-hospital mortality across range of 4C Mortality Score in validation cohort; lower panel: predicted versus observed probability of in-hospital mortality (calibration; red line) for 4C Mortality Score within validation cohort

Discrimination of the 4C Mortality Score in the validation cohort was similar to that of the XGBoost model (table 3). Calibration was also found to be excellent in the validation cohort: overall observed (30.1%) versus predicted (30.1%) mortality was equal (calibration-in-the-large=0) and calibration was excellent over the range of risk (slope=1, Brier score 0.171; fig 2). The 4C Mortality Score showed good performance in clinically relevant metrics across a range of cut-off values (table 4).

**Table 4:** Performance metrics of 4C Mortality Score to rule out and rule in mortality at different cut-off values in validation cohort

Cut-off value	No of patients (%)			TP		TN	FP	FN	Sensitivity (%)		Specificity (%)		PPV (%)		NPV (%)		Mortality (%)
**Rule out mortality**																	
≤2	1001 (4.5)			6724		996	14 636	5	99.9		6.4		31.5		99.5		0.5
≤3	1650 (7.4)			6709		1630	14 002	20	99.7		10.4		32.4		98.8		1.2
≤4	2420 (10.8)			6672		2363	13 269	57	99.2		15.1		33.5		97.6		2.4
≤6	4121 (18.4)			6542		3934	11 698	187	97.2		25.2		35.9		95.5		4.5
≤8	6539 (29.2)			6223		6033	9599	506	92.5		38.6		39.3		92.3		7.7
≤9	8167 (36.5)			5911		7349	8283	818	87.8		47		41.6		90.0		10.0
**Rule in mortality**																	
≥9		15 822 (70.8)	6223		6033		9599	506	92.5	38.6		39.3		92.3		39.3	
≥11		12 325 (55.1)	5483		8790		6842	1246	81.5	56.2		44.5		87.6		44.5	
≥13		8069 (36.1)	4206		11 769		3863	2523	62.5	75.3		52.1		82.3		52.1	
≥15		4158 (18.6)	2557		14 031		1601	4172	38	89.8		61.5		77.1		61.5	
≥17		1579 (7.1)	1142		15 195		437	5587	17	97.2		72.3		73.1		72.3	
≥19		381 (1.7)	305		15 556		76	6424	4.5	99.5		80.1		70.8		80.1	

FN=false negative; FP=false positive; NPV=negative predictive value; PPV=positive predictive value; TN=true negative; TP=true positive.

Four risk groups were defined with corresponding mortality rates determined (table 5): low risk (0-3 score, mortality rate 1.2%), intermediate risk (4-8 score, 9.9%), high risk (9-14 score, 31.4%), and very high risk (≥15 score, 61.5%). Performance metrics showed a high sensitivity (99.7%) and negative predictive value (98.8%) for the low risk group, covering 7.4% of the cohort and a corresponding mortality rate of 1.2%.

**Table 5:** Comparison of mortality rates for 4C Mortality Score risk groups across derivation and validation cohorts

Risk group	Derivation cohort			Validation cohort	
	No of patients (%)	No of deaths (%)		No of patients (%)	No of deaths (%)
Low (0-3)	2574 (7.3)	45 (1.7)		1650 (7.4)	20 (1.2)
Intermediate (4-8)	8277 (23.3)	751 (9.1)		4889 (21.9)	486 (9.9)
High (9-14)	18 091 (51.0)	6310 (34.9)		11 664 (52.2)	3666 (31.4)
Very high (≥15)	6521 (18.4)	4320 (66.2)		4158 (18.6)	2557 (61.5)
Overall	35 463	11 426		22 361	6729

Patients in the intermediate risk group (score 4-8, n=4889, 21.9%) had a mortality rate of 9.9% (negative predictive value 90.1%). Patients in the high risk group (score 9-14, n=11 664, 52.2%) had a mortality rate of 31.4% (negative predictive value 68.6%), while patients scoring 15 or higher (n=4158, 18.6%) had a mortality rate of 61.5% (positive predictive value 61.5%). An interactive infographic is available at https://isaric4c.net/risk

### Comparison with existing tools

**Fig 3:**
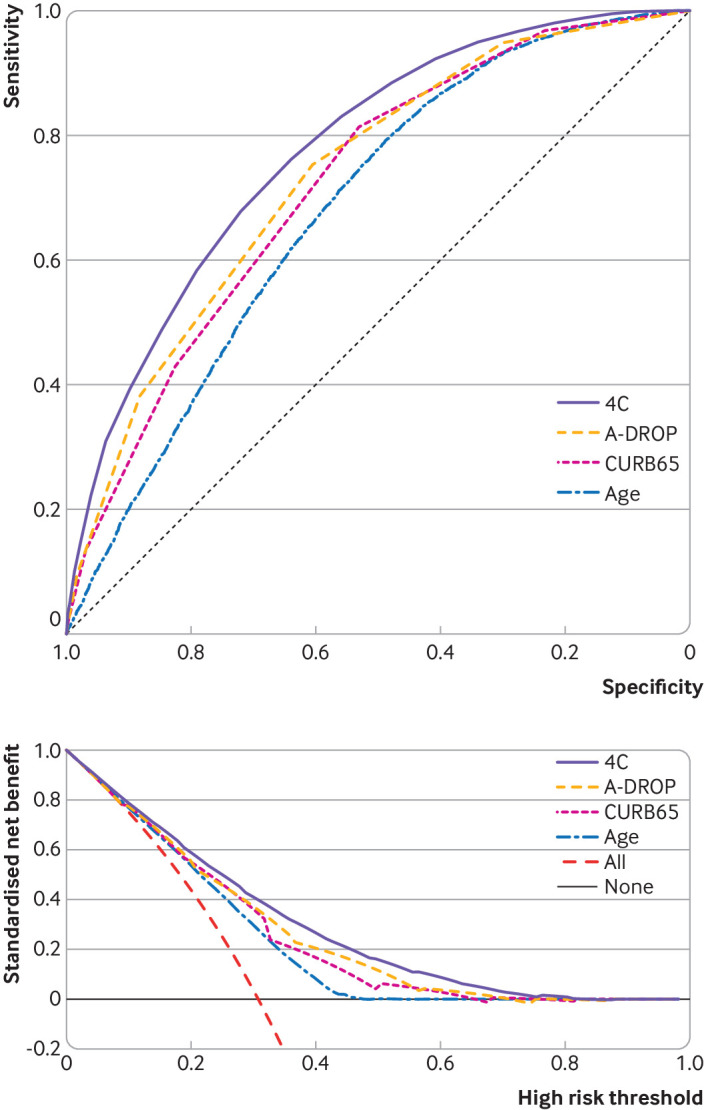
Receiver operator characteristic curves (upper panel) and decision curve analysis (lower panel) for most discriminating three models applicable to more than 50% of validation population compared with age alone (restricted cubic spline; imputed datasets). In lower panel, lines are shown for standardised net benefit at different risk thresholds of treating no patients (black line) and treating all patients (red dashed line)

We performed a systematic literature search and identified 15 risk stratification scores that could be applied to these data.^6^ ^22^ ^28^ ^29^ ^30^ ^31^ ^32^ ^33^ ^34^ ^35^ ^36^ ^37^ ^38^ ^39^ ^40^ The 4C Mortality Score compared well against these existing risk stratification scores in predicting in-hospital mortality (table 6, fig 3, upper panel). Risk stratification scores originally validated in patients with community acquired pneumonia (n=9) generally had higher discrimination for in-hospital mortality in the validation cohort (eg, A-DROP (area under the receiver operating characteristic curve 0.74, 95% confidence interval 0.73 to 0.74) and E-CURB65 (0.76, 0.74 to 0.79)) than those developed within covid-19 cohorts (n=4: Surgisphere (0.63, 0.62 to 0.64), DL score (0.67, 0.66 to 0.68), COVID-GRAM (0.71, 0.68 to 0.74), and Xie score (0.73, 0.70 to 0.75)). Performance metrics for the 4C Mortality Score compared well against existing risk stratification scores at specified cut-off values (appendix 13).

**Table 6:** Discriminatory performance of risk stratification scores within validation cohort (complete case) to predict in-hospital mortality in patients with covid-19

Model	Validation cohort*	
	No of patients with required parameters	AUROC (95% CI)
SOFA	197	0.614 (0.530 to 0.698)
qSOFA	19 361	0.622 (0.615 to 0.630)
Surgisphere†	18 986	0.630 (0.622 to 0.639)
SMARTCOP	486	0.645 (0.593 to 0.697)
NEWS	19 074	0.654 (0.645 to 0.662)
DL score†	16 345	0.669 (0.660 to 0.678)
SCAP	370	0.675 (0.620 to 0.729)
CRB65	19 361	0.683 (0.676 to 0.691)
COVID-GRAM†	1239	0.706 (0.675 to 0.736)
DS-CRB65	18 718	0.718 (0.710 to 0.725)
CURB65	15 560	0.720 (0.713 to 0.728)
Xie score†	1753	0.727 (0.701 to 0.753)
A-DROP	15 572	0.736 (0.728 to 0.744)
PSI	360	0.736 (0.683 to 0.790)
E-CURB65	1553	0.764 (0.740 to 0.788)
4C Mortality Score	14 398	0.774 (0.767 to 0.782)

AUROC=area under the receiver operating characteristic curve; covid-19=coronavirus disease 2019.

See appendix 13 for other metrics.

* Available data.

† Novel covid-19 risk stratification score.

The number of patients in whom risk stratification scores could be applied differed owing to certain variables not being available, either because of missingness or because they were not tested for or recorded in clinical practice. Seven scores could be applied to fewer than 2000 patients (<10%) in the validation cohort owing to the requirement for biomarkers or physiological parameters that were not routinely captured (eg, lactate dehydrogenase). Decision curve analysis showed that the 4C Mortality Score had better clinical utility across a wide range of threshold risks compared with the best performing existing scores applicable to more than 50% of the validation cohort (A-DROP and CURB65; fig 3, lower panel).

### Sensitivity analysis 

Sensitivity analyses that used complete case data showed similar discrimination (appendix 7) and performance metrics (appendices 8 and 9) to analyses that used the imputed dataset. After stratification of the validation cohort into two geographical cohorts (validation north and south; appendix 14), discrimination remained similar for the 4C Mortality Score in the north subset (area under the receiver operating characteristic curve 0.77, 95% confidence interval 0.76 to 0.78) and south subset (0.76, 0.75 to 0.77; appendix 6).

Finally, we checked discrimination of the 4C Mortality Score by sex and ethnic group (appendix 10). Discrimination was the same in men (area under the receiver operating characteristic curve 0.77, 95% confidence interval 0.76 to 0.78) and women (0.76, 0.75 to 0.77). Discrimination was better in all non-white ethnic groups compared with the white group: South Asian (0.82, 0.80 to 0.85), East Asian (0.85, 0.79 to 0.91), Black (0.83, 0.80 to 0.86), and other ethnic minority (0.81, 0.79 to 0.84).

## Discussion

### Principal findings

We have developed and validated the eight variable 4C Mortality Score in a UK prospective cohort study of 57 824 patients admitted to hospital with covid-19. The 4C Mortality Score uses patient demographics, clinical observations, and blood parameters that are commonly available at the time of hospital admission and can accurately characterise the population of patients at high risk of death in hospital. The score compared favourably with other models, including best-in-class machine learning techniques, and showed consistent performance across the validation cohorts, including good clinical utility in a decision curve analysis.

Model performance compared well against other generated models, with minimal loss in discrimination despite its pragmatic nature. A machine learning approach showed a marginal improvement in discrimination, but at the cost of interpretability, the requirement for many more input variables, and the need for an app or website calculator that might limit use at the bedside given personal protective equipment requirements. The 4C Mortality Score showed good applicability within the validation cohort and consistency across all performance measures.

### Comparison with other studies

The 4C Mortality Score contains parameters reflecting patient demographics, comorbidity, physiology, and inflammation at hospital admission; it shares characteristics with existing prognostic scores for sepsis and community acquired pneumonia but has important differences as well. No pre-existing score appears to use this combination of variables and weightings. Altered consciousness and high respiratory rate are included in most risk stratification scores for sepsis and community acquired pneumonia,^21^ ^22^ ^28^ ^29^ ^32^ ^33^ ^36^ while raised urea is also a common component.^21^ ^22^ ^28^ Increasing age is a strong predictor of in-hospital mortality in our cohort of patients admitted with covid-19 and is commonly included in other existing covid-19 scores,^37^ ^41^ ^42^ together with comorbidity^37^ ^41^ ^42^ and raised C reactive protein.^40^ ^43^

Discriminatory performance of existing covid-19 scores applied to our cohort was lower than reported in derivation cohorts (DL score 0.74, COVID-GRAM 0.88, Xie score 0.98).^37^ ^38^ ^40^ The use of small inpatient cohorts from Wuhan, China for model development might have resulted in overfitting, limiting generalisability in other cohorts.^38^ ^40^ The Xie score demonstrated the highest discriminatory power (0.73), and included age, lymphocyte count, lactate dehydrogenase, and peripheral oxygen saturations. However, we were only able to apply this score for less than 10% of the validation cohort because lactate dehydrogenase is not routinely measured on hospital admission in the UK.

Owing to challenges of clinical data collection during an epidemic, missing data are common, with choice of predictors influenced by data availability.^40^ Complete case analysis often leads to exclusion of a substantial proportion of the original sample, subsequently leading to a loss of precision and power.^44^ However, the assessment of missing data on model performance in novel covid-19 risk stratification scores has been limited^37^ or unexplored,^38^ ^40^ potentially introducing bias and further limiting generalisability to other cohorts. We found discriminatory performance in both derivation and validation cohorts remained similar after the imputation of a wide range of variables,^41^ further supporting the validity of our findings.

The presence of comorbidities is handled differently in covid-19 prognostic scores; comorbidities might be included individually,^40^ ^42^ given equal weight,^37^ or found to have no predictive effect.^38^ Recent evidence suggests an additive effect of comorbidity in patients with covid-19, with increasing number of comorbidities associated with poorer outcomes.^16^ In our cohort, the inclusion of individual comorbidities within the machine learning model conferred minimal additional discriminatory performance, supporting the inclusion of an overall comorbidity count.

### Strengths and limitations of this study

The ISARIC WHO CCP-UK study represents a large prospectively collected cohort admitted to hospital with covid-19 and reflects the clinical data available in most economically developed healthcare settings. We derived a clinically applicable prediction score with clear methods and tested it against existing risk stratification scores in a large patient cohort admitted to hospital. The score compared favourably with other prognostic tools, with good to excellent discrimination, calibration, and performance characteristics.

The 4C Mortality Score has several methodological advantages over current covid-19 prognostic scores. The use of penalised regression methods and an event-to-variable ratio greater than 100 reduce the risk of overfitting.^45^ ^46^ The use of parameters commonly available at first assessment increases its clinical applicability, avoiding the requirement for markers often only available after a patient has been admitted to a critical care facility.^4^ ^47^ Of course a model developed in a specific dataset should describe that dataset best. However, by including comparisons with pre-existing models, reassurance is provided that equivalent performance cannot be delivered with a simple tool already in use.

Additionally, in a pandemic, baseline infection rates and patient characteristics might change by time and geography. This motivated the temporal and geographical validation, which is crucial to the reporting of this study. These sensitivity analyses showed that score performance continued to be robust over time and geography.

Our study has limitations. Firstly, we were unable to evaluate the predictive performance of several existing scores that require a large number of parameters (for example, APACHE II^48^), as well as several other covid-19 prognostic scores that use computed tomography findings or uncommonly measured biomarkers.^5^ Additionally, several potentially relevant comorbidities, such as hypertension, previous myocardial infarction, and stroke,^16^ were not included in data collection. The inclusion of these comorbidities might have impacted upon or improved the performance and generalisability of the 4C Mortality Score.

Secondly, a proportion of recruited patients (3.3%) had incomplete episodes. Selection bias is possible if patients with incomplete episodes, such as those with prolonged hospital admission, had a differential mortality risk to those with completed episodes. Nevertheless, the size of our patient cohort compares favourably to other datasets for model creation. The patient cohort on which the 4C Mortality Score was derived comprised patients admitted to hospital who were seriously ill (mortality rate of 32.2%) and were of advanced age (median age 73 years). This model is not for use in the community and could perform differently in populations at lower risk of death. Further external validation is required to determine whether the 4C Mortality Score is generalisable among younger patients and in settings outside the UK.

## Conclusions and policy implications

We have derived and validated an easy-to-use eight variable score that enables accurate stratification of patients with covid-19 admitted to hospital by mortality risk at hospital presentation. Application within the validation cohorts showed this tool could guide clinician decisions, including treatment escalation.

A key aim of risk stratification is to support clinical management decisions. Four risk classes were identified and showed similar adverse outcome rates across the validation cohort. Patients with a 4C Mortality Score falling within the low risk groups (mortality rate 1%) might be suitable for management in the community, while those within the intermediate risk group were at lower risk of mortality (mortality rate 10%; 22% of the cohort) and might be suitable for ward level monitoring. Similar mortality rates have been identified as an appropriate cut-off value in pneumonia risk stratification scores (CURB-65 and PSI).^21^ ^22^ Meanwhile patients with a score of 9 or higher were at high risk of death (around 40%), which could prompt aggressive treatment, including the initiation of steroids^49^ and early escalation to critical care if appropriate.

**What is already known on this topic**

Robust, validated clinical prediction tools are lacking that identify patients with coronavirus disease 2019 (covid-19) who are at the highest risk of mortality
Given the uncertainty about how to stratify patients with covid-19, considerable interest exists in risk stratification scores to support frontline clinical decision making
Available risk stratification tools have a high risk of bias, small sample size resulting in uncertainty, poor reporting, and lack formal validation

**What this study adds**

Most existing covid-19 risk stratification tools performed poorly in our cohort; caution is needed when novel tools based on small patient populations are applied to cohorts in hospital with covid-19
The 4C (Coronavirus Clinical Characterisation Consortium) Mortality Score is an easy-to-use and valid prediction tool for in-hospital mortality, accurately categorising patients as being at low, intermediate, high, or very high risk of death
This pragmatic and clinically applicable score outperformed other risk stratification tools, showed clinical decision making utility, and had similar performance to more complex models

## Web extra

Extra material supplied by authors

Web appendix: Supplementary material

## Notes

Contributors: Conceptualisation: SRK, AH, RP, GC, JD, PWH, LM, JSN-V-T, CS, PJMO, JKB, MGS, ABD, EMH; data curation: RP, SH, KAH, CJ, CAG, KAM, LM, LN, CDR, CAS, MGS, ABD, EMH; formal analysis: SRK, AH, RP, TMD, CJF, KAM, MGP, LCWT, EMH. Funding acquisition: PWH, JSN-V-T, TS, PJMO, JKB, MGS, ABD. Investigation: SRK, AH, CDR, OVS, LCWT, PJMO, JKB, MGS, ABD, EMH; methodology: SRK, AH, RP, IB, KAM, CAS, AS, CS, ABD, EMH; project administration: SH, HEH, CJ, LM, LN, CDR, CAS, MGS; resources: CG, PWH, LM, TS, MGS, EMH; software: SRK, RP, KAM, OVS; supervision: SRK, RP, CG, HEH, PWH, PLO, PJMO, JKB, MGS, EMH; visualisation: SRK, RP, MGP, EMH; writing-original draft: SRK, EMH; writing-review and editing: SRK, RG, AH, RP, IB, GC, TMD, CAG, JD, CJF, LM, MN, JSN-V-T, PLO, MGP, CDR, AS, CS, OVS, LCWT, PJMO, JKB, MGS, ABD, EMH. SRK and AH are joint first authors. MGS, ABD, and EMH are joint senior authors. MGS is guarantor and corresponding author for this work, and attests that all listed authors meet authorship criteria and that no others meeting the criteria have been omitted.

Ethical approval: Ethical approval was given by the South Central-Oxford C Research Ethics Committee in England (reference 13/SC/0149), and by the Scotland A Research Ethics Committee (reference 20/SS/0028). The study was registered at https://www.isrctn.com/ISRCTN66726260.

Data sharing: We welcome applications for data and material access via our Independent Data and Material Access Committee (https://isaric4c.net).

The lead author (the manuscript’s guarantor) affirms that the manuscript is an honest, accurate, and transparent account of the study being reported; that no important aspects of the study have been omitted; and that any discrepancies from the study as planned (and, if relevant, registered) have been explained.

Dissemination to participants and related patient and public communities: ISARIC-4C has a public facing website isaric4c.net and twitter account (@CCPUKstudy). We are engaging with print and internet press, television, radio, news, and documentary programme makers. We will explore distribution of findings with The Asthma UK and British Lung Foundation Partnership and take advice from NIHR Involve and GenerationR Alliance Young People’s Advisory Groups.

Provenance and peer review statement: Not commissioned; externally peer reviewed.
