# Risk stratification of patients admitted to hospital with covid-19 using the ISARIC WHO Clinical Characterisation Protocol: development and validation of the 4C Mortality Score

**DOI:** 10.1136/bmj.m4334

**Published:** 2020-11-13

In this paper by Knight and colleagues (*BMJ* 2020;370:m3339, doi:10.1136/bmj.m3339, published 9 September 2020), the following corrections apply:

- The legend to figure 2 should read: “Upper panel: distribution of patients across range of 4C Mortality Score in validation cohort; middle panel: observed in-hospital mortality across range of 4C Mortality Score in validation cohort; lower panel: predicted versus observed probability of in-hospital mortality (calibration; red line) for 4C Mortality Score within the validation cohort.”
- In table 2, the first urea cut-off value should be “<7” instead of “≤7”, and the units for C reactive protein should be “mg/L” instead of “mg/dL.”

The online article and PDF will be updated in due course.

