# Supplementary material for: Risk stratification of patients admitted to hospital with covid-19 using the ISARIC WHO Clinical Characterisation Protocol: development and validation of the 4C Mortality Score
Source: BMJ. 2020 Sep 9;370:m3339. doi: 10.1136/bmj.m3339 (PMC7116472; doi:10.1136/bmj.m3339)
Supplement: Supplementary file 1 — Web appendix: Supplementary material [file knir060903.ww1.pdf]

Supplementary material

Appendix 1. ISARIC WHO CCP-UK risk stratification score derivation and validation protocol

Background

Patients hospitalised with covid-19 are at high risk of mortality. Stratification of patients on admission may aid clinicians in determining immediate management decisions (home discharge, ward-level care, escalation to ICU) and medical treatment. High risk of bias exists in novel covid-19 risk stratification tools, with small cohorts in limited geographical areas and potential for over-fitting. Many of these scores are also complex to calculate. This limits clinical utility.

Aims

- Develop risk stratification score using the largest known hospitalised cohort of covid-19 patients
- The risk stratification score must have high clinical utility (defined here as having the ability to be calculated without a complex equation or algorithm)
- Following derivation, determine discriminatory performance in validation cohorts and compare to existing risk stratification tools (for pneumonia, influenza and covid-19)

Primary outcome

In-hospital mortality with minimum 28d follow up.

Patient inclusion

- All adult patients (≥18 years old on admission)
- Index admission (readmission episode excluded)
- Completed index admission

Potential candidate variables

Identification

The systematic literature search (see below) will identify potential predictor variables for mortality, disease severity and/or critical care requirement in pneumonia, influenza or covid-19 patients.

Inclusion criteria

- Readily available patient or clinical characteristic to attending clinicians upon presentation to hospital (Accident & Emergency department, Acute Medical Receiving Unit)
- Blood markers should be commonly measured and results available for review within the first 24 hours of admission
- Measured within the ISARIC WHO CCP-UK database (pre-specified case report form)

Exclusion criteria

- Variable with missing values in >33% of patients within the derivation cohort

Score development

All patients within the database on 20th May 2020 will be included within the derivation cohort.

With the overall aim to derive a risk stratification score with high clinical utility, an *a priori* decision has been made to categorise final included predictor variables for ease of calculation in a clinical environment. However, to avoid loss of information through categorisation, generalised additive models (GAMs) will be used first to identify final predictor variables prior to categorisation.

All remaining candidate predictor variables following application of exclusion criteria (availability in database, missingness) will be entered into a GAMs. These variables will then be removed individually and GAMs run again, determining the explained deviance and unbiased risk estimator (UBRE; essentially a scaled AIC) following exclusion of each individual variable. Final variables to include within the risk stratification score will then be selected by explained deviance, R^2 and UBRE.

GAMs curves for each continuous variable will then be created for each final included variable and cut-offs determined based on outcome risk. Once categorised, the final variables will be placed in a least absolute shrinkage and selection operator (LASSO) to ensure all final variables should be selected within the risk score and to reduce the risk of over-fitting. Shrunk coefficients will be converted to produce an index score.

In parallel, a machine learning (ML) model will be derived using extreme gradient boosted trees methodology (XGBoost), representing a 'best-in-class' model. This will include all candidate predictor variables included within the GAM model.

Statistical analysis

Model performance will be determined using the AUROC, with calibration and Brier score calculated for each final model (derived risk score and ML model).

To determine the impact of missingness, a missing data analysis will be performed. Multivariate imputation by chained equations (MICE) will be used for all candidate predictor variables (except those with high levels of missingness). It will be assumed that variables are missing at random and the primary outcome will be used in derivation dataset imputation models. Ten sets with ten iterations will be performed. Model performance will again be determined as detailed above, with Rubin's Rules used to combine model parameter estimates.

All statistical analysis will use the R (v3.6.3).

Validation

All patients entered into the database after the specified derivation cohort cut-off will be included, with the same patient inclusion/criteria applied.

Exiting risk stratification tool identification

Risk stratification scores created and or validated for pneumonia, influenza and covid-19 will be included. These will be identified through the systematic literature search (see below) and do not have to have been peer-reviewed for inclusion.

Only risk stratification scores with all predictor variables will be considered for inclusion. Decisions for inclusion of risk stratification score where one variable is missing will be made on a case-by-case basis by consensus within the study group. If the missing variable is deemed a key contributor to risk prediction within the tool it will be excluded.

Statistical analysis

Discriminatory performance (AUROC) and other performance metrics (sensitivity, specificity, PPV and NPV) will be calculated for all included risk stratification tools and compared with the derived risk and ML model in each of the validation cohorts. Calibration and Brier score will also be determined for the derived risk score in each validation cohort.

Systematic literature search

Databases

EMBASE, WHO Medicus and Google Scholar (particularly for pre-print publications)

Search terms

Pneumonia; sepsis; influenza; covid-19; SARS-CoV-2; coronavirus;

Combined with: score and prognosis

No language or date restrictions

Appendix 2. Candidate predictor variables evaluated for potential inclusion in modelling process

	Evidence and/or models	Inclusion / exclusion
**Patient demographics**		
Age on admission (years)	CURB-65[a], COVID-GRAM[b]	Included
Sex at Birth	A-DROP[c], PSI[d]	Included
Ethnicity	Ethnicity predicts clinical outcomes in covid-19[e]	Included
Hypertension	Comorbidity predicts clinical outcomes in covid-19[f]	Excluded – not initially recorded within the ISARIC CCP-UK database
Chronic cardiac disease	Comorbidity predicts clinical outcomes in covid-19[f]	Combined with other comorbidities for model development
Chronic kidney disease	Comorbidity predicts clinical outcomes in covid-19[f]	Combined with other comorbidities for model development
Malignant neoplasm	Comorbidity predicts clinical outcomes in covid-19[f]	Combined with other comorbidities for model development
Moderate or severe liver disease	Comorbidity predicts clinical outcomes in covid-19[f]	Combined with other comorbidities for model development
Obesity (clinician defined)	Comorbidity predicts clinical outcomes in covid-19[f]	Combined with other comorbidities for model development
Chronic pulmonary disease (not asthma)	Comorbidity predicts clinical outcomes in covid-19[f]	Combined with other comorbidities for model development
Diabetes (type 1 & type 2)	Comorbidity predicts clinical outcomes in covid-19 [f]	Combined with other comorbidities for model development
Number of comorbidities	Number of comorbidities predicts clinical outcomes in covid-19[f]	Included – composite count of all included comorbidities defined by Charlson Comorbidity Index plus obesity
**Clinical signs/ observations**		
Respiratory Rate	CURB65[a], NEWS2[g]	Included
Peripheral oxygen saturations (%)	Xie score[h], ADROP[c]	Included
Systolic blood pressure (mmHg)	CURB-65[a], NEWS2[g]	Included
Diastolic blood pressure (mmHg)	CURB-65[a]	Included
Temperature (°C)	PSI[d], NEWS2[g]	Included
Heart Rate (bpm)	NEWS2[g]	Included
Glasgow Coma Score	COVID-GRAM[b], CURB-65[a]	Included
**Bedside investigations**		
FiO2	NEWS2[g], SOFA[i]	Excluded – too many values missing from derivation dataset
PaO2 (kPa)	PSI[d], SCAP[j]	Excluded – too many values missing from derivation dataset
pH	PSI[d], SCAP[j]	Excluded – too many values missing from derivation dataset

	Evidence and/or models	Inclusion / exclusion
Glucose (mmol/L)	PSI[d]	Excluded – too many values missing from derivation dataset
Infiltrates on chest radiograph	COVID-GRAM[b] , PSI[d], SMART-COP[k]	Excluded – too many values missing from derivation dataset
**Laboratory measures**		
Haemoglobin (g/L)	Severe covid-19 known to lower haemoglobin concentration[l]	Included
White cell count (10^9/L)	COVID-GRAM[b]	Included
Neutrophil count (10^9/L)	COVID-GRAM[b], DL score[m]	Included
Lymphocyte count (10^9/L)	COVID-GRAM[b]	Included
Haematocrit (%)	PSI[d]	Excluded – too many values missing from derivation dataset
Platelet Count (10^9/L)	DL score[m], E-CURB65[n]	Included
Prothrombin (seconds)	Coagulopathy associated with mortality in covid-19 patients[o]	Excluded – too many values missing from derivation dataset
Activated partial thromboplastin time (APTT) (seconds)	Coagulopathy associated with mortality in covid-19 patients[o]	Excluded – too many values missing from derivation dataset
Sodium (mmol/L)	PSI[d]	Included
Total Bilirubin (mg/dL)	COVID-GRAM[b], SOFA[i]	Included
Alanine aminotransferase (ALT) (units/L)	Abnormal liver tests associated with severe covid-19[p]	Excluded – too many values missing from derivation dataset
Aspartate aminotransferase (AST) (units/L)	Abnormal liver tests associated with severe covid-19[p]	Excluded – too many values missing from derivation dataset
Albumin (g/L)	Association between low albumin and severe covid-19[q]	Excluded - not recorded within ISARIC CCP-UK database
Lactate dehydrogenase (Units/L)	COVID-GRAM[b], E-CURB65[n]	Excluded – too many values missing from derivation dataset
Urea (mmol/L)	CURB-65[a], A-DROP[c], PSI[d]	Included
Creatinine (µmol/L)	SOFA[i]	Included
C-reactive protein (CRP; mg/dL)	Associated with poorer outcomes in patients with covid-19[r, s]	Included

[a]CURB65 (abbreviation representing included model variables)
[b]COVID-GRAM (Liang JAMA Int Med 2020)
[c]A-DROP (abbreviation representing included model variables)
[d]Pneumonia Severity Index (PSI)
[e]Pan 2020. EClin Med; doi: https://doi.org/10/1016/j.eclinm.2020.100404
[f]Guan 2020. Eur Respir J. May; 55(5): 2000547
[g]National Early Warning Score (NEWS2)
[h]Xie score (Xie MedRxiv 2020)
[i]Sequential Organ Failure Assessment (SOFA) score
[j]Severe Community Acquired Pneumonia (SCAP) score
[k]SMART-COP (abbreviation representing included model variables)
[l]Lippi G et al. Hematol Transfus Cell Ther. 2020

[m]DL score (Zhang MedRxiv 2020)
[n]Expanded CURB65 score (E-CURB65)
[o]Zhou 2020, Lancet 395; P1054-1062
[p]Cai 2020, DOI: https://doi.org/10.1016/j.jhep.2020.04.006
[q]Aziz 2020, Critical Care. 24:255
[r]Liu 2020, European Respiratory Journal; 55(5): 2001112
[s]Luo 2020, Clin Infect Dis; doi: https//doi.org/10.1093/cid/ciaa641

Appendix 3. Criterion-based approach using generalised additive models for remaining 20 candidate variables following exclusion for missing values. Inclusion criteria specified as >1% change in deviance explained (>0.2) or >10% change in the unbiased risk estimator (>0.002) compared to the full GAM model containing all candidate variables.

Variable	Deviance explained (%)	Reduction in deviance explained on removal from 21 variable model (%)	R^2	Unbiased Risk Estimator (UBRE)	Area under receiver operator cure	Included in final model
**All candidate variables**	**23.3**	**-**	**0.261**	**-0.030**	**0.811**	**-**
Age	19.1	4.2	0.221	0.022	0.786	Included
Sex at Birth	23.1	0.2	0.260	-0.029	0.811	Included
Ethnicity	23.2	0.1	0.261	-0.030	0.811	
Number of comorbidities	22.9	0.4	0.258	-0.026	0.809	Included
Respiratory Rate	22.8	0.5	0.256	-0.025	0.809	Included
Peripheral oxygen saturations	22.2	1.1	0.249	-0.017	0.805	Included
Systolic blood pressure	23.2	0.0	0.261	-0.030	0.811	
Diastolic blood pressure	23.2	0.0	0.261	-0.030	0.811	
Temperature	23.2	0.0	0.261	-0.030	0.811	
Heart Rate	23.1	0.1	0.260	-0.029	0.810	
Glasgow Coma Score	22.9	0.4	0.257	-0.025	0.809	Included
Haemoglobin	23.1	0.1	0.260	-0.029	0.810	
White cell count	23.2	0.0	0.261	-0.030	0.811	
Neutrophil count	23.2	0.0	0.261	-0.030	0.811	
Lymphocyte count	23.1	0.1	0.260	-0.029	0.809	
Platelet Count	23.1	0.1	0.260	-0.029	0.810	
Sodium	23.2	0.0	0.261	-0.030	0.811	
Total Bilirubin	23.2	0.0	0.261	-0.030	0.811	
Urea	22.7	0.6	0.256	-0.024	0.808	Included
Creatinine	23.2	0.0	0.261	-0.030	0.811	
C-reactive protein	22.7	0.6	0.256	-0.024	0.808	Included
**Final eight variable model**	**21.2**	**2.1**	**0.241**	**-0.009**	**0.798**	**-**

Appendix 4. Continuous smoothed predictors (thin-plate splines) for numerical variables generated from primary generalised additive model (GAM). Linearity of response was assessed (log-odds scale) and the location of gradient changes determined. The methods of Barrio *et al*[15] were used to identify cut points on the basis of slopes and the clinical significance of the cut point in question.

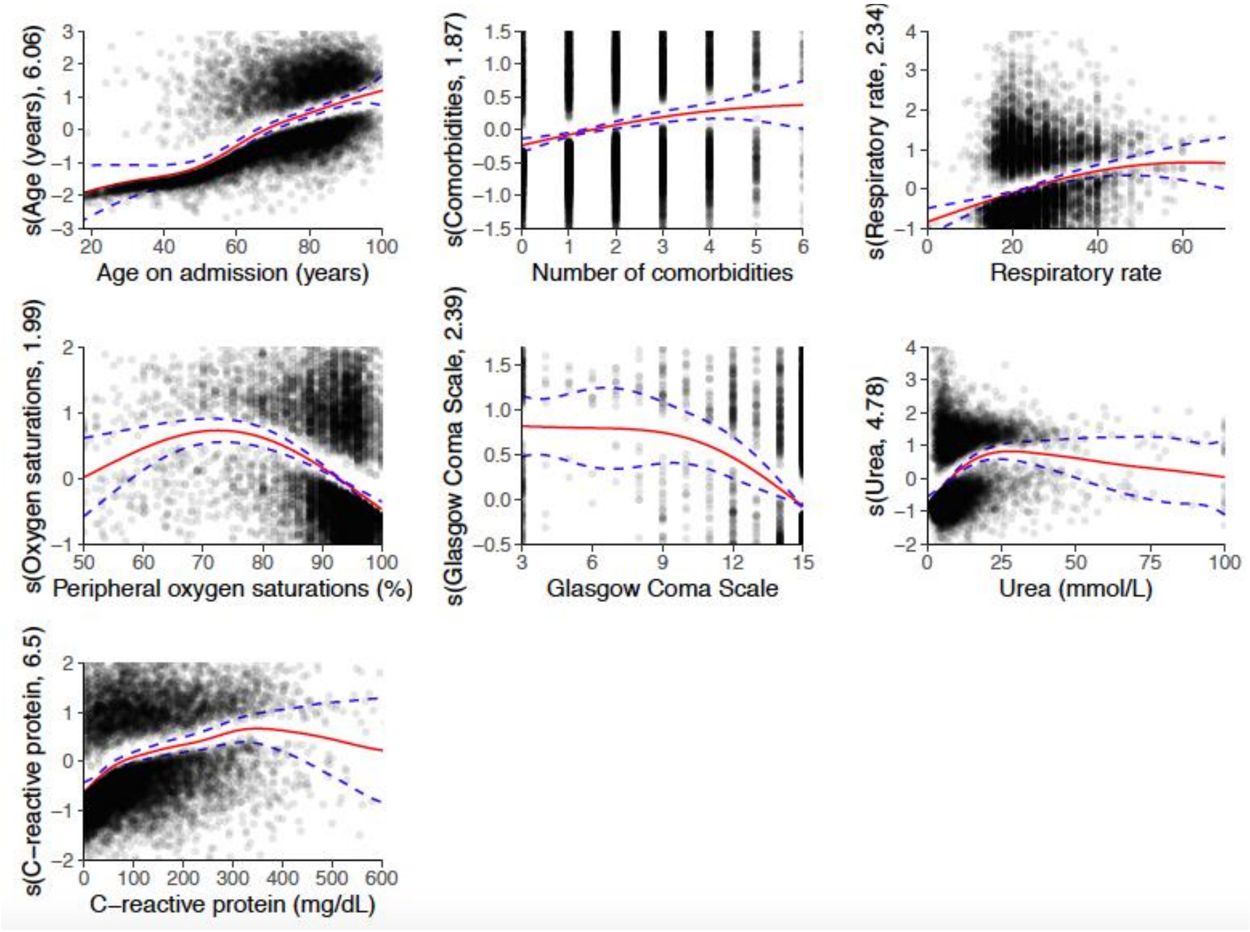

Appendix 5. A, penalised regression coefficients from LASSO logistic regression (log-odds scale) which were scaled to produce the prognostic index. B, regression coefficients (y-axis) at different values of L1 penalty (x-axis) for 4C Mortality Score.

A

	Level	Penalised coefficient	Penalised coefficient (x3 scaling)
Intercept	-	-4.203	-13
Age (years)	50-59	0.687	2
	60-69	1.337	4
	70-79	1.842	6
	≥80	2.252	7
Sex at birth	Male	0.172	1
Number of comorbidities*	1	0.300	1
	≥2	0.532	2
Respiratory rate (breaths/minute)	20-29	0.232	1
	≥30	0.649	2
Oxygen saturation on room air (%)	<92	0.577	2
Glasgow Coma Scale	<15	0.558	2
Urea (mmol/L)	7-14	0.439	1
	>14	1.011	3
CRP (mg/dL)	50-99	0.363	1
	≥100	0.74	2

*Comorbidities were defined using the Charlson Comorbidity Index, with the addition of clinician-defined obesity

B

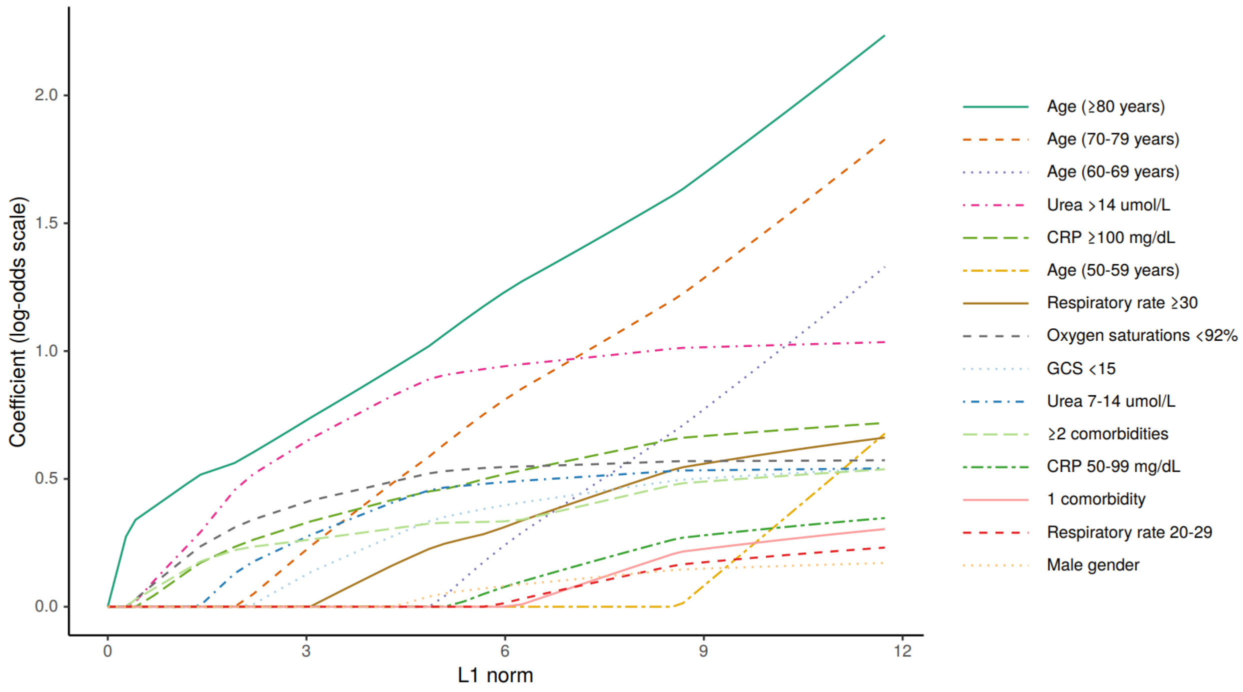

Coefficient (log-odds scale)
2.0
1.5
1.0
0.5
0.0
0
3
6
9
12
L1 norm
Age (≥80 years)
Age (70-79 years)
Age (60-69 years)
Urea >14 umol/L
CRP ≥100 mg/dL
Age (50-59 years)
Respiratory rate ≥30
Oxygen saturations <92%
GCS <15
Urea 7-14 umol/L
≥2 comorbidities
CRP 50-99 mg/dL
1 comorbidity
Respiratory rate 20-29
Male gender

Appendix 6. Discrimination of models in imputed validation dataset. Missing data patterns were analysed (finalfit package) and data were considered missing at random (as opposed to missing completely at random). Multiple imputation of missing values was performed (mice package) with 10 iterations to create 10 imputed sets using the 28 predictor variables plus outcome for the derivation cohort, and 28 predictors without outcome in the validation cohort. Imputation methods were continuous variables: predictive mean matching; 2-level factors: binary logistic regression; and >2-level factors: polytomous regression (all considered unordered). Distributions of imputed variables were inspected across iterations.

Generalised additive model (GAM) included continuous predictors with L2-penalised thin-plate splines with comorbidities considered as a continuous count.

Gradient boosting tree (XGBoost) models included all continuous predictors and categorical predictors, including individual comorbidities. Two models were trained. The first used the multiply imputed datasets. The second used non-imputed data with missing modelled in the model building process.

Penalised logistic regression (LASSO) model used categorised variables with discrimination determined using exact coefficients.

4C mortality score is the prognostic index developed from the model building process.

	Variable N	Derivation AUROC (95% CI)	Validation AUROC (95% CI)	Validation North AUROC (95% CI)	Validation South AUROC (95% CI)
GAM	21	0.798 (0.793 – 0.803)	0.774 (0.767 – 0.780)	0.781 (0.773 – 0.789)	0.763 (0.752 – 0.774)
XGBoost (imputed)	28	0.796 (0.786 – 0.807)	0.779 (0.772 – 0.785)	0.785 (0.777 – 0.793)	0.769 (0.758 – 0.779)
XGBoost (missing modelled)	28	0.786 (0.775 – 0.797)	0.782 (0.776 – 0.788)	0.789 (0.781 – 0.797)	0.774 (0.763 – 0.785)
LASSO	8	0.788 (0.783 – 0.793)	0.768 (0.761 – 0.774)	0.772 (0.764 – 0.781)	0.761 (0.750 – 0.771)
4C mortality	8	0.786 (0.781 – 0.790)	0.767 (0.760 – 0.773)	0.771 (0.763 – 0.779)	0.760 (0.749 – 0.770)

GAM, generalized additive model; XGBoost, gradient boosting decision tree; LASSO, penalised logistic regression; AUROC, area under receiver operator characteristic curve; CI, confidence interval.

Appendix 7. Sensitivity analysis: discrimination of models with complete case data.

	Variable N	Derivation complete	Validation complete
GAM	21	0.800 (0.794 – 0.806)	0.783 (0.776 – 0.791)
XGBoost (imputed)	28	0.772 (0.761 – 0.783)	0.782 (0.776 – 0.789)
LASSO	8	0.789 (0.783 –0.795)	0.776 (0.769 – 0.784)
4C mortality	8	0.786 (0.780 – 0.792)	0.774 (0.767 – 0.782)

GAM, generalized additive model; XGBoost, gradient boosting decision tree; LASSO, penalised logistic regression; AUROC, area under receiver operator characteristic curve; CI, confidence interval.

Appendix 8. Sensitivity analysis with complete case data: Performance metrics of 4C Mortality Score to rule-out mortality (A) and rule-in mortality (B) at different cut-offs in validation cohort.

A

	Number of patients at cut-off (%)	TP	TN	FP	FN	Sensitivity	Specificity	PPV	NPV	Mortality (%)
<=2	539 (3.7)	4588	537	9271	2	100.0	5.5	33.1	99.6	0.4
<=3	955 (6.6)	4583	948	8860	7	99.8	9.7	34.1	99.3	0.7
<=4	1428 (9.9)	4558	1396	8412	32	99.3	14.2	35.1	97.8	2.2
<=6	2532 (17.6)	4478	2420	7388	112	97.6	24.7	37.7	95.6	4.4
<=8	4044 (28.1)	4296	3750	6058	294	93.6	38.2	41.5	92.7	7.3
<=9	5003 (34.7)	4115	4528	5280	475	89.7	46.2	43.8	90.5	9.5

B

	Number of patients at cut-off (%)	TP	TN	FP	FN	Sens	Spec	PPV	NPV	Mortality (%)
>=9	10354 (71.9)	4296	3750	6058	294	93.6	38.2	41.5	92.7	41.5
>=11	8244 (57.3)	3835	5399	4409	755	83.6	55	46.5	87.7	46.5
>=13	5590 (38.8)	3012	7230	2578	1578	65.6	73.7	53.9	82.1	53.9
>=15	3019 (21.0)	1913	8702	1106	2677	41.7	88.7	63.4	76.5	63.4
>=17	1185 (8.2)	868	9491	317	3722	18.9	96.8	73.2	71.8	73.2
>=19	277 (1.9)	221	9752	56	4369	4.8	99.4	79.8	69.1	79.8

TP, true positive; TN, true negative; FP, false positive; FP, false positive; PPV, positive predictive value; NPV, negative predictive value.

Appendix 9. Sensitivity analysis with complete case data: Comparison of mortality rates for 4C Mortality Score risk groups across derivation and validation cohorts.

	Derivation cohort		Validation cohort	
Risk group	Number of patients (%)	Mortality (%)	Number of patients (%)	Mortality (%)
Low (0-3)	1472 (6.9)	22 (1.5)	955 (6.6)	7 (0.7)
Intermediate (4-8)	4873 (22.7)	429 (8.8)	3089 (21.5)	287 (9.3)
High (9-14)	10 795 (50.3)	3779 (35.0)	7335 (50.9)	2383 (32.5)
Very high (≥15)	4305 (20.1)	2816 (65.4)	3019 (21.0)	1913 (63.4)
Overall	21 445	7046	14 142	4590

Appendix 10. Sensitivity analysis with imputed datasets: Discrimination of 4C Mortality Score by ethnicity and sex.

		N	Derivation AUROC (95% CI)	N	Validation AUROC (95% CI)
Sex at birth	Male	20678	0.785 (0.779-0.791)	12164	0.768 (0.759-0.776)
	Female	14785	0.786 (0.778-0.793)	10197	0.764 (0.754-0.773)
Ethnicity	White	29030	0.778 (0.773-0.783)	18924	0.757 (0.750-0.764)
	South Asian	1868	0.823 (0.803-0.843)	951	0.823 (0.795-0.851)
	East Asian	303	0.808 (0.757-0.859)	175	0.851 (0.790-0.912)
	Black	1443	0.817 (0.793-0.840)	871	0.827 (0.796-0.857)
	Other Ethnic Minority	2819	0.803 (0.785-0.820)	1440	0.813 (0.789-0.838)

AUROC, area under receiver operator characteristic curve; N, number of patients; CI, confidence interval.

Appendix 11. Calibration plot of 4C Mortality Score in derivation cohort.

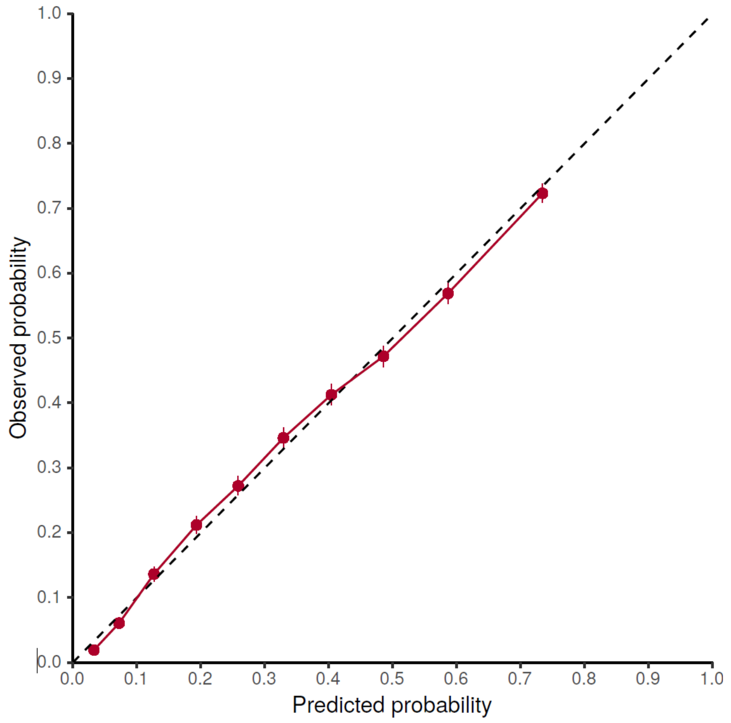

Appendix 12. Components of included risk stratification scores (*indicates novel covid-19 risk score).

A

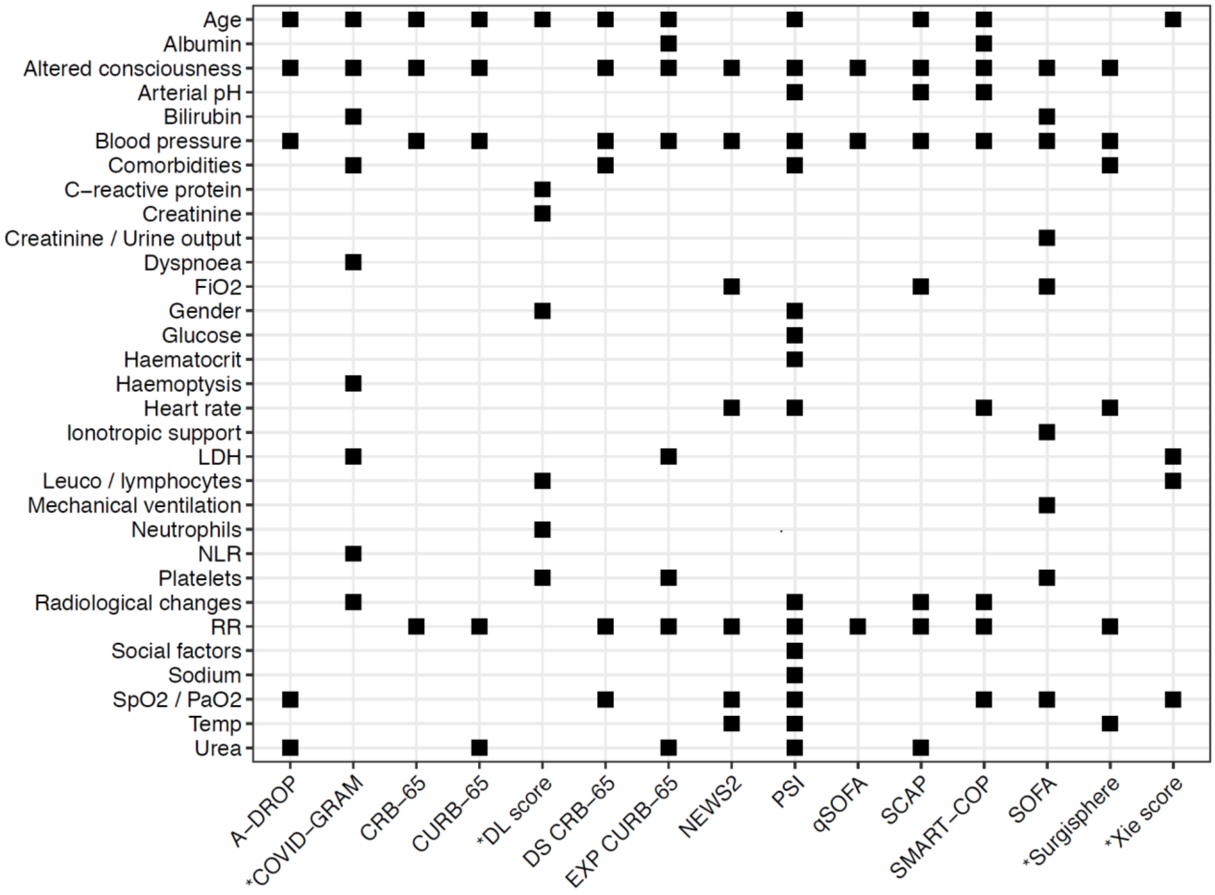

B

Score	Condition	Outcome
A-DROP (Miyashita Int Med 2006)	Community-acquired pneumonia	30-day mortality
COVID-GRAM (Liang JAMA Int Med 2020)	covid-19	Mortality and/or ICU admission
CRB65 (Bauer J Int Med 2006)	Community-acquired pneumonia	30-day mortality
CURB65 (Lim Thorax 2003)	Community-acquired pneumonia	30-day mortality
DL score (Zhang MedRxiv 2020)	covid-19	Mortality / ICU admission
DS-CRB65 (Dwyer BMJ ORR 2014)	Community-acquired pneumonia	30-day mortality
E-CURB65 (Liu Sci Rep 2016)	Community-acquired pneumonia	30-day mortality
NEWS2 (Royal College of Physicians, UK 2012)	Sepsis	In-hospital mortality
PSI (Fine NEJM 1997)	Community-acquired pneumonia	Low risk of 30-day mortality
qSOFA (Singer JAMA 2016)	Sepsis	In-hospital mortality
SCAP (Yandiola Chest 2009)	Community-acquired pneumonia	Adverse outcome*
SMART-COP (Charles Clin Infect Dis 2008)	Community-acquired pneumonia	Need for ventilator or vasopressor support
SOFA (Vincent Int Care Med 1996)	Sepsis	ICU mortality
Surgisphere (no definitive publication)	covid-19	In-hospital mortality / critical illness**
Xie score (Xie MedRxiv 2020)	covid-19	Mortality

*ICU admission, need for mechanical ventilation, severe sepsis, or treatment failure)

**Measured outcome unclear in online material

Appendix 13. Operative characteristics of included risk stratification scores to predict mortality at reported cut-offs within validation cohorts.

Test	TP	TN	FP	FN	Sensitivity	Specificity	PPV	NPV
Surgisphere (>4)	4328	5597	7242	1526	73.9	43.6	37.4	78.6
Surgisphere (>7)	2544	9646	3193	3310	43.5	75.1	44.3	74.5
qSOFA (>1)	1346	12 015	1100	4601	22.6	91.6	55.0	72.3
qSOFA (>2)	198	13 041	74	5749	3.3	99.4	72.8	69.4
NEWS2 (>4)	3090	9014	3914	2760	52.8	69.7	44.1	76.6
NEWS2 (>6)	1736	11 259	1669	4114	29.7	87.1	51.0	73.2
SMART-COP (>2)	129	115	216	24	84.3	34.7	37.4	82.7
SMART-COP (>4)	49	281	50	104	32.0	84.9	49.5	73.0
SMART-COP (>6)	10	320	11	143	6.5	96.7	47.6	69.1
SCAP (≥10)	163	36	159	9	94.8	18.5	50.6	80.0
SCAP (≥20)	96	138	57	76	55.8	70.8	62.7	64.5
SCAP (≥30)	42	184	11	130	24.4	94.4	79.2	58.6
DL score (Low)	4020	4986	5892	1160	77.6	45.8	40.6	81.1
DL score (High)	3441	6462	4416	1739	66.4	59.4	43.8	78.8
CRB65 (=0)	5599	3636	9479	347	94.2	27.7	37.1	91.3
CRB65 (>2)	861	12 630	485	5085	14.5	96.3	64.0	71.3
DS-CRB65 (>1)	4815	6037	6618	965	83.3.	47.7	42.1	86.2
DS-CRB65 (>2)	2832	10 079	2576	2948	49.0	79.6	52.4	77.4
DS-CRB65 (>3)	1148	12 045	610	4632	19.9	95.2	65.3	72.2
CURB65 (>1)	4004	5537	4861	919	81.3	53.3	45.2	85.8
CURB65 (>2)	2092	8640	1758	2831	42.5	83.1	54.3	75.3
CURB65 (>3)	648	10 092	306	4275	13.2	97.1	67.9	70.2
A-DROP (≥3)	1864	9234	1175	3060	37.9	88.7	61.3	75.1
A-DROP (≥4)	448	10 236	173	4476	9.1	98.3	72.1	69.6
E-CURB65 (>2)	361	653	408	94	79.3	61.5	46.9	87.4
E-CURB65 (>4)	67	1030	31	388	14.7	97.1	68.4	72.6
PSI (>70)	123	44	185	5	96.1	19.2	39.9	89.8
PSI (>90)	113	91	138	15	88.3	39.7	45.0	85.8
PSI (>130)	73	172	57	55	57.0	75.1	56.2	75.8

Some included scores did not provide cut-off values. TP – True positive; TN - True negative; FP – False positive; FP – False positive; PPV – Positive predictive value; NPV – Negative predictive value. *Derived in covid-19 cohort

Appendix 14. Demographic and clinical characteristics for validation cohort after stratification by geography.

		Validation North dataset (n = 13 769)	Validation South dataset (n = 8592)
Number of hospitals included		117	86
Mortality (%)		4287 (31.1)	2442 (28.4)
Age (years)	<50	1599 (11.6)	1210 (14.1)
	50-59	1561 (11.3)	1058 (12.3)
	60-69	1877 (13.6)	1266 (14.7)
	70-79	3198 (23.2)	1753 (20.4)
	≥80	5534 (40.2)	3305 (38.5)
Sex at Birth	Male	7359 (53.6)	4782 (55.7)
	Female	6380 (46.4)	3798 (44.3)
Ethnicity	White	11 098 (89.1)	5732 (77.9)
	South Asian	4234 (3.5)	377 (5.1)
	East Asian	54 (0.4)	86 (1.2)
	Black	279 (2.2)	490 (6.7)
	Other Ethnic Minority	590 (4.7)	677 (9.2)
Chronic cardiac disease		4548 (35.1)	2471 (32.3)
Chronic kidney disease		2453 (19.1)	1316 (17.4)
Malignant neoplasm		1387 (10.9)	800 (10.6)
Moderate or severe liver disease		294 (2.3)	140 (1.9)
Obesity (clinician defined)		1412 (12.3)	822 (12.1)
Chronic pulmonary disease (not asthma)		2499 (19.4)	1238 (16.3)
Diabetes (type 1 & type 2)		2659 (21.6)	1616 (22.4)
Number of comorbidities	0	2333 (17.6)	1583 (20.1)
	1	3732 (28.1)	2381 (30.2)
	≥2	7227 (54.3)	3921 (49.7)
Respiratory Rate		20.0 (7.0)	21.0 (8.0)
Oxygen saturation (%)		94.0 (5.0)	94.0 (6.0)
Systolic blood pressure (mmHg)		128.0 (33.0)	129.0 (33.0)
Diastolic blood pressure (mmHg)		73.0 (20.0)	73.0 (20.0)
Temperature (°C)		37.1 (1.5)	37.1 (1.5)
Heart Rate (bpm)		90.0 (27.0)	90.0 (27.0)
Glasgow Coma Score		15.0 (0.0)	15.0 (0.0)
Haemoglobin (g/L)		127.0 (31.0)	127.0 (31.0)
White cell count (10^9/L)		7.5 (5.3)	7.8 (5.4)
Neutrophil count (10^9/L)		5.7 (4.9)	6.0 (5.0)
Lymphocyte count (10^9/L)		0.9 (0.7)	0.9 (0.7)

	Validation North dataset (n = 13 769)	Validation South dataset (n = 8592)
Platelet Count (10^9/L)	222.0 (125.2)	224.0 (128.0)
Sodium (mmol/L)	137.0 (7.0)	137.0 (6.0)
Potassium (mmol/L)	4.1 (0.8)	4.1 (0.7)
Total Bilirubin (mg/dL)	9.0 (8.0)	10.0 (7.0)
Urea (mmol/L)	7.4 (6.7)	7.3 (6.9)
Creatinine (µmol/L)	86.0 (57.0)	87.0 (56.0)
C-reactive protein (CRP) (mg/dL)	75.0 (115.0)	82.0 (127.0)

Appendix 14. Sensitivity analysis of discriminatory performance for risk stratification scores after stratification of validation cohort by geography to predict inpatient mortality in patients hospitalised with covid-19.

	Validation North (N = 13 769)		Validation South (N = 8592)	
	N	AUROC (95% CI)	N	AUROC (95% CI)
SOFA	42	0.620 (0.443-0.797)	155	0.611 (0.514-0.707)
qSOFA	12250	0.624 (0.614-0.634)	7111	0.622 (0.609-0.635)
SMARTCOP	206	0.629 (0.554-0.704)	6984	0.624 (0.610-0.638)
Surgisphere*	12001	0.635 (0.624-0.645)	7009	0.642 (0.628-0.657)
SCAP	189	0.661 (0.584-0.738)	6355	0.659 (0.644-0.673)
NEWS	12064	0.662 (0.651-0.672)	7110	0.681 (0.669-0.693)
DL score*	9989	0.676 (0.665-0.687)	181	0.689 (0.610-0.769)
CRB65	12250	0.685 (0.676-0.694)	280	0.694 (0.623-0.765)
COVID-GRAM*	522	0.704 (0.659-0.749)	6875	0.709 (0.696-0.721)
CURB65	9815	0.723 (0.713-0.733)	716	0.709 (0.667-0.752)
DS-CRB65	11841	0.723 (0.714-0.732)	5743	0.717 (0.704-0.730)
Xie score*	688	0.737 (0.697-0.777)	1064	0.727 (0.693-0.761)
A-DROP	9814	0.740 (0.730-0.750)	5756	0.729 (0.716-0.742)
PSI	212	0.742 (0.675-0.810)	148	0.738 (0.649-0.827)
E-CURB65	621	0.778 (0.742-0.813)	931	0.757 (0.725-0.789)
**4C Mortality Score**	**8938**	**0.778 (0.768-0.788)**	**5458**	**0.769 (0.756-0.782)**
Machine learning comparison (XGBoost)	-	0.785 (0.777-0.793)	-	0.769 (0.758-0.779)

Appendix 15. Risk of bias assessment using PROBAST checklist.

Domain	Item		Development	ROB	Validation	ROB
Participants	1.1	Were appropriate data sources used, e.g. cohort, RCT or nested case-control study data?	Prospective longitudinal cohort design. Prespecified research database and predefined follow-up (hospital inpatient). Protocol for model development specified *a priori*	+	Prospective longitudinal cohort design. Prespecified research database and predefined follow-up (hospital inpatient)	+
	1.2	Were all inclusions and exclusions of participants appropriate?	All patients within cohort included	+	All patients within cohort included	+
Predictors	2.1	Were predictors defined and assessed in a similar way for all participants?	Predictors defined and assessed in the same way for all study participants	+	Predictors defined and assessed in the same way for all study participants	+
	2.2	Were predictor assessments made without knowledge of outcome data?	Prospective cohort design - prognostic predictors were assessed at time of admission and before outcome occurrence	+	Prospective cohort design - prognostic predictors were assessed at time of admission and before outcome occurrence	+
	2.3	Are all predictors available at the time the model is intended to be used?	All predictors available within the first few hours of admission to hospital	+	All predictors available within the first few hours of admission to hospital	+
Outcome	3.1	Was the outcome determined appropriately?	Objective outcome (mortality) used	+	Objective outcome (mortality) used	+
	3.2	Was a pre-specified or standard outcome definition used?	Yes - mortality	+	Yes - mortality	+
	3.3	Were predictors excluded from the outcome definition?	Outcome determined without information about predictors	+	Outcome determined without information about predictors	+
	3.4	Was the outcome defined and determined in a similar way for all participants?	Outcome (mortality) defined and determined in the same way for all study participants	+	Outcome (mortality) defined and determined in the same way for all study participants	+
	3.5	Was the outcome determined without knowledge of predictor information?	Outcome determined without information about predictors	+	Outcome determined without information about predictors	+
	3.6	Was the time interval between predictor assessment and outcome determination appropriate?	Time interval between predictor measurement and outcome clinically appropriate (in-hospital mortality)	+	Time interval between predictor measurement and outcome clinically appropriate (in-hospital mortality)	+
Analysis	4.1	Were there a reasonable number of participants with the outcome?	High events per variable (>20)	+	High events per variable (>20) and >100 events overall	+

	4.2	Were continuous and categorical predictors handled appropriately?	Continuous variables examined for nonlinearity using thin-plate splines. Optimal cut-points were selected using the methods of Barrio et al.	+	Use of same predictors and scale as derived model	+
	4.3	Were all enrolled participants included in the analysis?	All participants included in the analysis	+	All participants included in the analysis	+
	4.4	Were participants with missing data handled appropriately?	Multiple imputation methods (MICE) used for missing data	+	Multiple imputation methods (MICE) used for missing data	+
	4.5	Was selection of predictors based on univariable analysis avoided?	Univariable analysis not performed	+	N/A	+
	4.6	Were complexities in the data (e.g. censoring, competing risks, sampling of controls) accounted for appropriately?	Model developed using a full cohort approach and short-term follow-up (inpatient mortality)	+	Full cohort approach used and short-term follow-up (inpatient mortality)	+
	4.7	Were relevant model performance measures evaluated appropriately?	Model calibration and discrimination (AUROC) assessed, together with classification measures	+	Model calibration and discrimination (AUROC) assessed, together with classification measures	+
	4.8	Were model overfitting and optimism in model performance accounted for?	10-fold cross-validation performed using least absolute shrinkage and selection operator (LASSO) logistic regression	+	N/A	+
	4.9	Do predictors and their assigned weights in the final model correspond to the results from multivariable analysis?	Predictors and regression coefficients of the final developed model, including intercept, are fully reported and correspond with the 4C Mortality Score index values	+	N/A	+

*ROB – Risk of Bias; + indicates low ROB/low concern regarding applicability; − indicates high ROB/high concern regarding applicability; and ? indicates unclear ROB/unclear concern regarding applicability.
